# Targeting ferroptosis with natural products in stroke: therapeutic mechanisms and translational opportunities

**DOI:** 10.3389/fphar.2025.1586345

**Published:** 2025-05-29

**Authors:** Yingyi Zheng, Xi Tan, Xiaojie Wang, Rui Mao, Jianwen Guo

**Affiliations:** ^1^ The Second Clinical College of Guangzhou University of Chinese Medicine, Guangzhou, China; ^2^ The Second Affiliated Hospital of Guangzhou University of Chinese Medicine, Guangzhou, China

**Keywords:** natural products, stroke, ferroptosis, Phytomedicine, intracerebral hemorrhage, ischemic stroke

## Abstract

Ferroptosis is a new type of controlled cell death. It is distinguished by its reliance on iron and the production of lipid peroxidation. The role of ferroptosis in stroke has attracted a lot of attention recently. The purpose of this review is to clarify the connection between ferroptosis and stroke and to investigate the potential contribution of natural products to the clinical management of stroke and the discovery of novel medications. In this review, we summarize in detail the mechanism of ferroptosis after stroke, especially the relevant targets of ferroptosis after stroke. Furthermore, we summarize the natural products and herbal medicine currently employed in ferroptosis along with their mechanisms of action, highlighting the potential and challenges of clinical translation. We included 55 articles and classified them. After systematic screening, We think that ginkgolide B, kellerin, loureirin C, quercetin, icariside II, salvianolic acid A, berberine, Dl-3-n-butylphthalide is an effective candidate drug for the treatment of stroke.

## 1 Introduction

Stroke, which contributes to a growing portion of the global medical burden, can be broadly classified into ischemic stroke and hemorrhagic stroke, with the latter incorporating intracerebral hemorrhage (ICH) and subarachnoid hemorrhage (SAH) (GBD, 2021 Diseases and Injuries Collaborators, 2024). Ischemic stroke is characterized by brain, spinal cord, or retinal infarction, representing approximately 85% of all strokes globally (Tsao et al., 2023). In accordance with the secondary stroke prevention guidelines established by the American Heart Association/American College of Cardiology, antiplatelet medications, including aspirin, clopidogrel, and dipyridamole, have become the primary therapeutic alternative (Medina-Inojosa et al., 2023). Nevertheless, a substantial problem has emerged: certain patients exhibit resistance to aspirin, which could potentially undermine the efficacy of these medications (Venketasubramanian et al., 2022). Given this predicament, it is imperative to create novel therapeutic agents that are effective for individuals who are resistant to conventional remedies.

**FIGURE 1:**
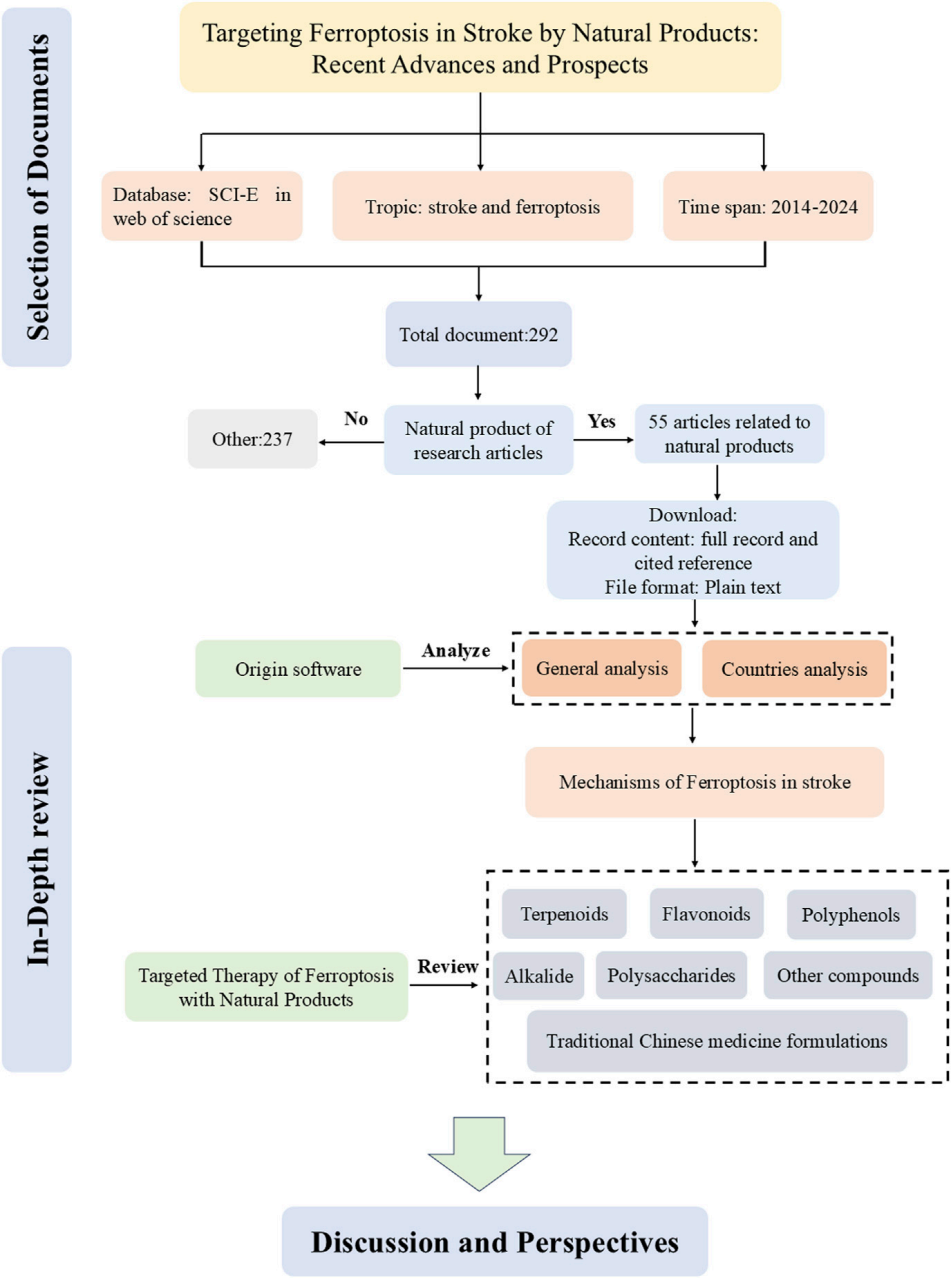
The flow chart of review.

Ferroptosis, a type of programmed cell death associated with stroke, was initially identified in 2012 and displays distinctive features, such as changes in the structure and density of mitochondria (Dixon et al., 2012; Xu et al., 2023a). Thus, a complete understanding of the function of ferroptosis in stroke could serve to offer new intervention targets. Natural products are the metabolites or plant metabolites of insects, microbes, marine, plant, and animal extracts, as well as many more endogenous chemical plant metabolites and metabolites found in human and animal bodies (Xing et al., 2023). They are proven to be a valuable source of new drugs (Liang et al., 2021). Many natural products have shown good therapeutic effects on stroke (Liu et al., 2023a). In this review, we summarize the mechanisms of ferroptosis in stroke and in detail the natural products of anti-ferroptosis therapy. Figure 1 shows a flowchart summarizing natural products that target ferroptosis after strokes.

## 2 Research trends on ferroptosis in stroke

**FIGURE 2:**
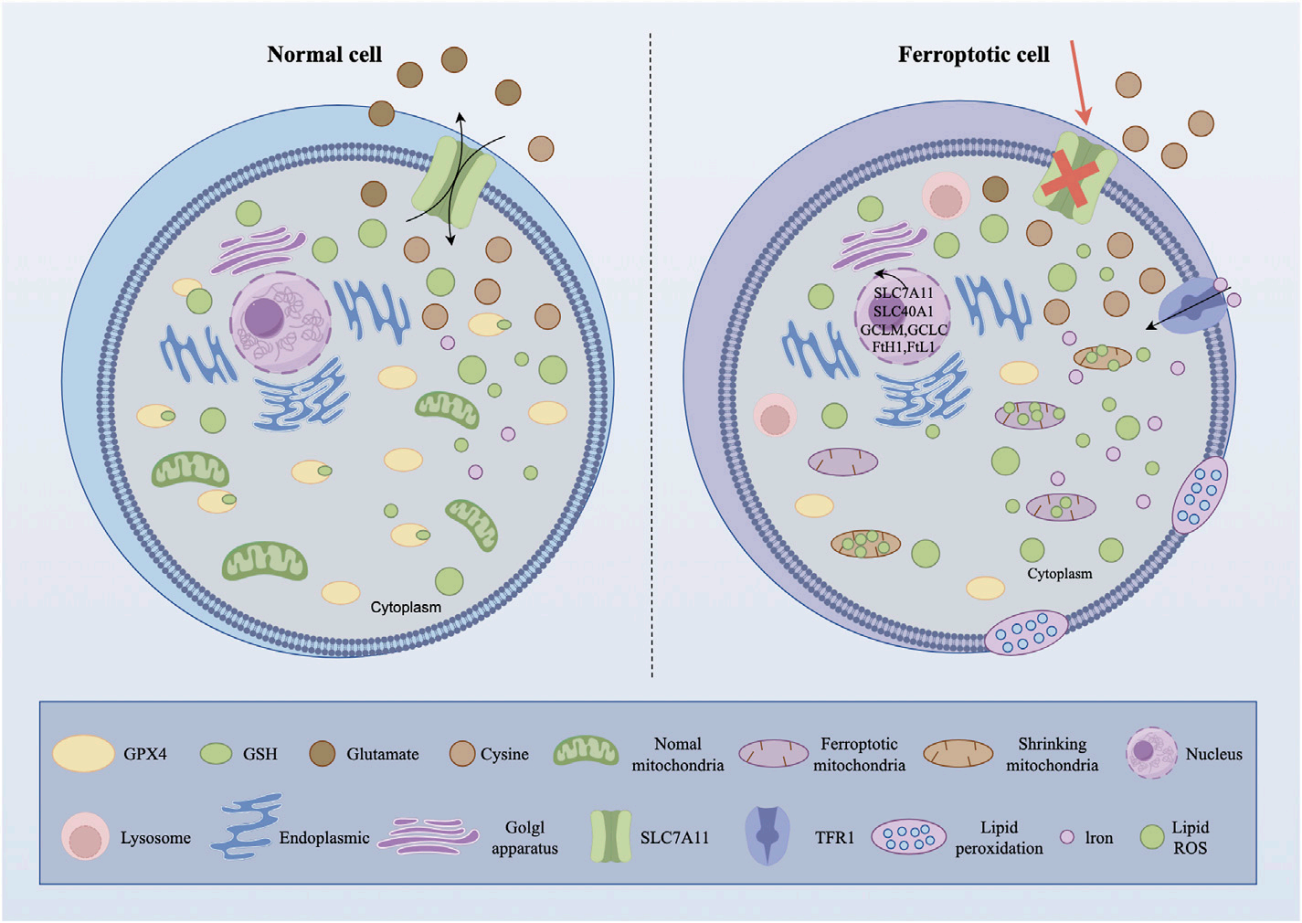
Ferroptosis causes damage to cells (figure made by figdraw).

Ferroptosis is a novel form of regulatory cell death associated with the formation of iron-dependent reactive oxygen species (ROS) and lipid peroxides (Dixon et al., 2012). Disordered iron metabolism in cells leads to the generation of excessive iron ions, which produce a significant quantity of ROS via the Fenton reaction (Dixon et al., 2012). ROS target polyunsaturated fatty acids (PUFAs) within the lipid membrane, generating lipid peroxides, compromising the integrity of the cell membrane, disrupting mitochondrial activity, and ultimately resulting in ferroptosis (Dixon et al., 2012). Figure 2 illustrates the distinction between ferroptosis cells and normal cells.

**FIGURE 3:**
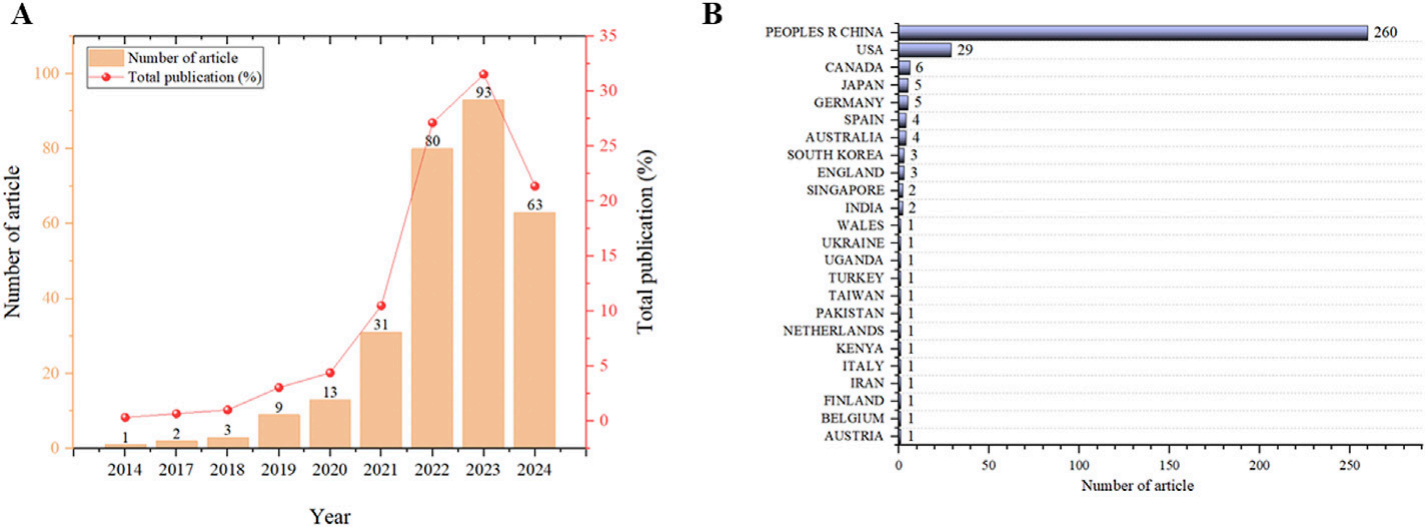
Scientometric study of ferroptosis on stroke. **(A)** Number of publications by year. **(B)** Number of publications by country or region.

The terms, “ferroptosis” and “stroke” as topic, were searched in Web of Science (SCI-E) core collection database. The article was refined and used for scientometric analysis. The time span is from 1 January 2014, to 31 December 2024. Original articles related to natural products were included in our study after being read in full by the researchers. The first paper, published in 2014, was titled “(−)-Epicatechin protects hemorrhagic brain via synergistic Nrf2 pathways” (Figure 3A). Most papers were published in the year 2023, while the most cited year was 2021. So far, research in the field of the therapeutic on the ferroptosis of stroke on natural products is increasingly ongoing. A total of 24 countries contributed to the publications included in this study. Among them, China published the most papers, followed by American, Canada, Japan, Germany, Spain, Australia, South Korea, England, and Singapore (Figure 3B). Next, we conducted a thorough review of these 293 articles, and 55 of them were included in our study (Figure 1).

## 3 Mechanisms of ferroptosis in ICH

**FIGURE 4:**
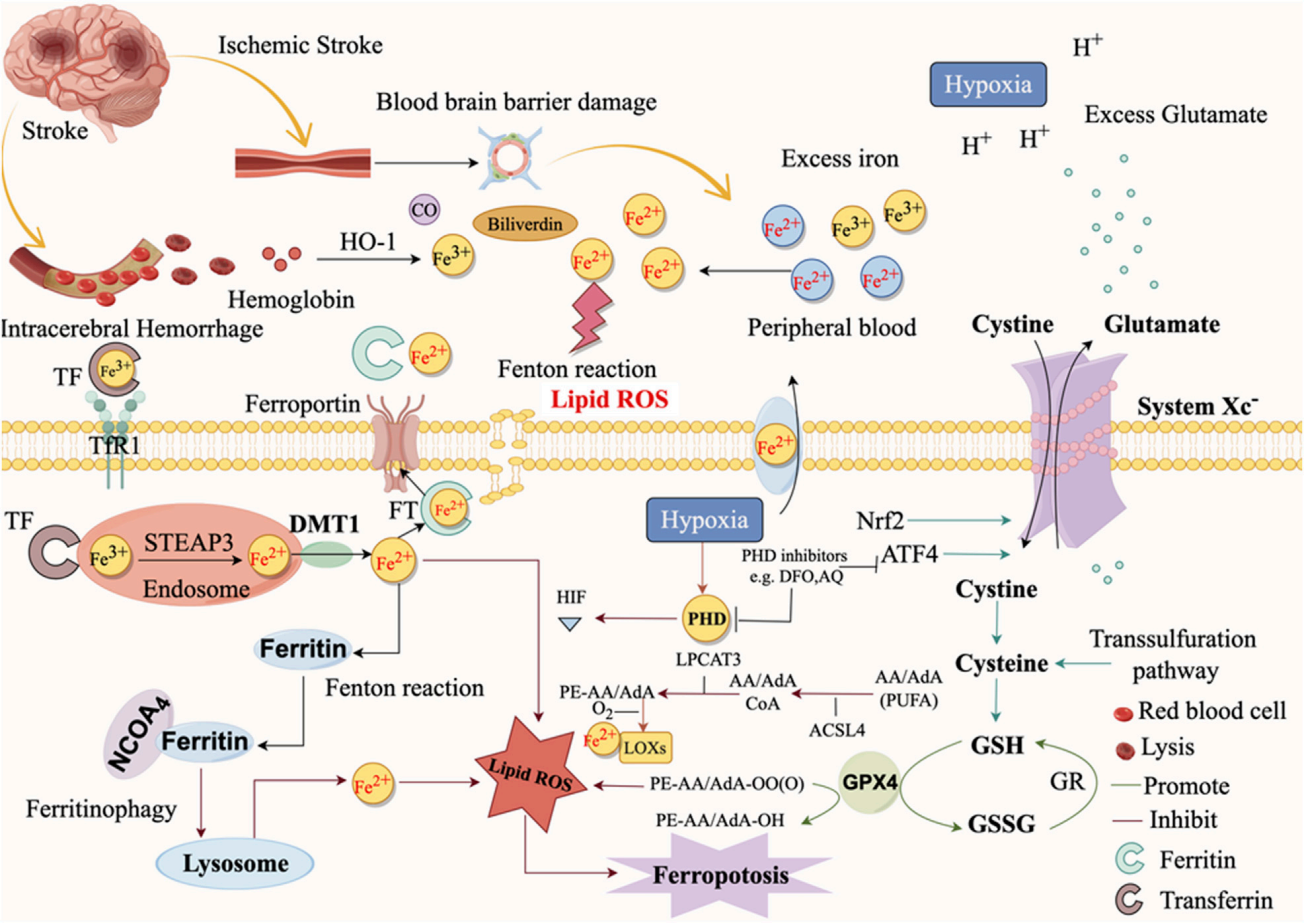
Mechanisms of ferroptosis after stroke (figure made by figdraw).

The accumulation of iron ions produced by red blood cells (RBCs) lysis after ICH is the main cause of ferroptosis (Figure 4). The lysis of RBCs in the hematoma is the most contributor of free hemoglobin, followed by the release of heme, which is subsequently degraded into iron, biliverdin, and carbon monoxide (Zhang et al., 2021). Iron ions accumulate after 24 h of ICH, and excessive accumulation is an important characteristic of ferroptosis (Sun et al., 2022). Under physiological conditions, the plasma membrane’s transferrin receptor TFR1 internalizes to facilitate transport (Zhao et al., 2021a). The iron output protein FPN1, which couples with multicopper iron oxidase (like ceruloplasmin) on the plasma membrane, mediates the intracellular iron output (Zhao et al., 2021a). Alternatively, multivesicular and extracellular vesicles containing ferritin can also excrete iron out of the cell (Zhao et al., 2021a). Under ICH conditions, iron is transported to the extracellular space through the iron export protein FPN in microglia, causing a large amount of toxic iron to enter the brain tissue (Hvidberg et al., 2005).

To be specific, two-molecule Fe^3+^ participates in the transport of iron by binding to one molecule of transferrin, which transports Fe^3+^ to the intracellular by binding to the membrane protein transferrin receptor 1 (TFR1) on the surface of neurons to form a Tf-Fe^3+^-TFR1 complex. Fe^3+^ is then reduced to Fe^2+^ by the Six-Transmembrane Epithelial Antigen of Prostate 3 (STEAP3). Endosomes release Fe^2+^ into the cytoplasmic labile iron pool (LIP), which requires DMT1/Solute Carrier Family 11 Member 2 (SLC11A2) regulation (Chen et al., 2020; Yao et al., 2021; Zhou et al., 2020) (Figure 3). The synthesis of the essential enzyme lipoxygenases (LOXs) in lipid peroxidation (LPO) can be facilitated by Fe^2+^ in the LIP, which leads to ferroptosis. Furthermore, Fe^2+^ will engage in the Fenton reaction with hydrogen peroxide to generate hydroxyl radicals, which will in turn damage cellular plant metabolites and induce ferroptosis (Wan et al., 2019). ROS can be generated by iron-catalyzed enzymes, which in turn can induce lipid auto-oxidation, thereby promoting ferroptosis (Chen et al., 2020). Nuclear receptor coactivator 4 (NCOA4) also mediates ferritinophagy by binding to ferritin and subsequently transporting iron-bound ferritin to the autophagosome for lysosomal degradation and iron release. NCOA4 knockdown can prevent LPO and ferroptosis by reducing the amount of iron in the intracellular LIP (Fang et al., 2021). Through NCOA4, autophagy-related 5 (ATG5) and autophagy-related 7 (ATG7) promote ferroptosis by degradation of ferritin (Hou et al., 2016). The expression of TFR1 can be stimulated by nitrogen fixation 1 (NFS1), an iron-sulfur cluster biosynthetic enzyme, to improve ferroptosis (Alvarez et al., 2017). Further, heat shock factor-binding Protein 1 (HSPB1) is highly inducible after treatment with Erastin. Once activated, HSBP1 reduces iron levels by inhibiting TFR1 expression (Sun et al., 2015).

LPO is a critical mechanism that directly initiates ferroptosis. It is the process by which oxygen binds to lipids to generate lipid peroxides by forming peroxyl radicals. The Fenton reaction, which is initiated by iron stimulation, generates lipid ROS, which subsequently induces LPO and ultimately results in ferroptosis (Bai et al., 2019). Furthermore, the oxidation and esterification of PUFAs generate lipid peroxides, which in turn induce LPO (Yang et al., 2016). LPO can attack cells by destroying proteins, DNA, and lipid membranes and activating ferroptosis (Salvador, 2010). Inhibiting LPO has been a critical and effective strategy for safeguarding ICH by reducing ferroptosis. We have summarized the related genes of ferroptosis after ICH, as shown in Table 1.

**TABLE 1:** Genes associated with ferroptosis after ICH.

S.N.	Gene name	Cell/Animal model	Pathway/Axis	Ref
1	Nrf2	SD, autologous blood	Nrf2/ARE-GPX4	Duan et al. (2022)
2	HDAC1/2	C57, autologous blood BV2 HT22, hemin	Nrf2/HO1	Jiang et al. (2024)
3	SOX10	HT22, hemin	miR-29a-3p/ACSL4	Chen et al. (2023)
4	ACSL4	C57, collagenase VII-S HT22, hemin	HOTAIR/UPF1/ACSL4	Jin et al. (2021)
5	lncRNA H19	BMVECs, hemin	miR-106b-5p/ACSL4	Chen et al. (2021a)
6	GPX4	SD, collagenase VII BV2, hemoglobin	AMPK/mTORC1	Xie et al. (2023)
7	H3K9me3	C57, collagenase VII N2A/SK-N-SH, hemin	Suv39h1-mediated H3K9me3, repress Tfr1	Lan et al. (2023)
8	Nedd4	C57, collagenase VII HT22, hemin	Nedd4/DMT1	Lv et al. (2024)
9	FOXO3	C57, collagenase VII HT22, hemin	FOXO3/NOX4	Qin et al. (2022)
10	CISD2	C57, collagenase VII	AKT/mTOR	Li et al. (2023a)
11	H_2_S	BV2, hemin	CBS/H2S	Yu et al. (2023)
12	circAFF1	C57, collagenase VII Neurons, Hemoglobin	miR-140-5p/GSK-3β Wnt/β-catenin	Yin et al. (2022)
13	SLC7A11	HT-22, FAC	miR-122-5p/TP53/SLC7A11	Zhao et al. (2021b)
14	METTL3	C57, collagenase VII BMVECs, hemin/OGD	N6-methyladenosine/GPX4	Zhang et al. (2022a)
15	FSP1	C57, autologous blood	GPX4 pathway	Wang et al. (2022a)
16	Fpn	Fpn-floxed mice autologous blood	miR-124/Fpn	Bao et al. (2020)
17	IRP	C57, collagenase VII Neurons, hemin	miR-19b-3p	Yi and Tang (2021)

## 4 Mechanisms of ferroptosis in ischemic stroke

Following ischemic stroke, the BBB becomes compromised because of the breakdown of tight junction protein (Figure 4). This leads to the release of Fe^3+^ from the blood into the brain tissue, facilitated by TF and TFR1 (Zhao et al., 2023a). Afterwards, there is an excessive amount of Fe^2+^ which is then decreased and moved to the cytoplasm. ROS, which are generated quickly via the Fenton reaction, facilitate the degradation of nucleic acids, proteins, and membranes, leading to the occurrence of ferroptosis (Figure 4). When cerebral ischemia reperfusion occurs, the release of excitatory amino acids, represented by Glu, increases and accumulates in the synaptic cristae. The uptake of glutamate within cells reduces while the release of glutamate outside of cells increases, resulting in the inhibition of the system Xc^−^ (Xu et al., 2023a). Some studies have found through metabolomics that gamma-glutamyl dipeptide or tripeptide was found in the cell under Cys deprivation. These metabolites reduced the level of Glu and alleviated the sensitivity to ferroptosis (Kagan et al., 2017). But on the other hand, for cysteine-deprived cells, the catabolism of Gln promoted the synthesis of PUFA, and its decomposition products such as α-ketoglutaric acid could also increase the accumulation of lipid peroxides. These effects of glutamine restored the sensitivity of cells to cysteine-deficient ferroptosis (Gao et al., 2019). It is important to mention that while Gln can be broken down into Glu by the enzymes GLS1 and GLS2, only the catalysis of GLS2 is linked to ferroptosis (Gao et al., 2015).

Prior to the proposal of the notion of ferroptosis, there have been multiple instances of abnormal buildup of iron in ischemic brain tissue (Dietrich and Bradley, 1988; Kondo et al., 1995). Research indicates that neurological damage following an ischemic stroke is associated with dysregulation of brain iron metabolism and transport (Selim and Ratan, 2004). The impaired BBB facilitates the entry of circulating iron into brain tissue following an ischemic stroke, while neurons also experience an increase in iron uptake (Bu et al., 2021). The early extravasation of circulating transferrin into ischemic brain parenchyma is induced by cerebral ischemia-reperfusion (I/R), which also activates NF-κB and promotes the expression of the DMT1 1B subtype, thereby enhancing iron uptake (DeGregorio-Rocasolano et al., 2018). In addition to facilitating iron uptake, cerebral ischemia also increases intracellular iron storage. The concentration of ferritin in the plasma and cerebral spinal fluid of individuals with ischemic stroke increases within 24 h. This rise is linked to early neurological impairment and might potentially result in hemorrhagic transformation and severe brain oedema following tPA thrombolysis (Liu, 2021). Interestingly, researchers found that ferritin overexpression attenuated Tau phosphorylation and ROS production, reversed the decreases of glutathione (GSH) and SLC7A11 in rats’ hippocampal neurons after middle cerebral artery occlusion (MCAO), and had a protective effect on motor injury and memory deficits (Chen et al., 2021b). We have summarized the related genes of ferroptosis after ischemic stroke, as shown in Table 2.

**TABLE 2:** Targets associated with ferroptosis after ischemic stroke.

S.N.	Targets name	Model	Effect on ischemic stroke	Ref
1	Tau	C57, SD MCAO	Tau suppression prevents ferroptosis by iron-targeting interventions	Tuo et al. (2017)
2	LncRNA PVT1	PC12, C57 MCAO	PVT1 regulated ferroptosis through miR-214-mediated TFR1 and TP53 expression	Lu et al. (2020)
3	SSAT1	C57; tMCAO	The activation of SSAT1/ALOX15 axis may aggravate ischemic stroke injury via triggering neuronal ferroptosis	Zhao et al. (2022)
4	Thrombin	Human blood N27 cell, OGD/R C57, SD, MCAO	Thrombin-ACSL4 axis ameliorate ferroptotic neuronal injury	Tuo et al. (2022)
5	UBIAD1	Primary cell OGD/R SD, MCAO	UBIAD1 improved brain tissue impairment and neuronal death	Huang et al. (2022)
6	PGE2	Human samples	Inhibit iron accumulation and LPO	Xu et al. (2022)
7	FtMt	C57, MCAO	Ameliorate inflammation and hepcidin-mediated decreases in ferroportin1	Wang et al. (2021)
8	USP14	Primary cell, OGD/R C57, MCAO	Inhibition of USP14 reduces NCOA4 expression and protects neurons from ferroptosis	Li et al. (2021)
9	SIRT6	HT-22, OGD/R C57, MCAO	FTH1 and NCOA4 via SIRT6 influence ferritin autophagy	Yu et al. (2024)
10	mTOR	SD, MCAO	mTOR/SREBP1 Pathway inhibit ferroptosis	Lang et al. (2024)
11	ACSL4	PC12, OGD/R SD, MCAO	Inhibits ferroptosis by facilitating ACSL4 ubiquitination degradation	Ou et al. (2024)

## 5 Targeted therapy of ferroptosis with natural products

Whether it is ischemic stroke or ICH, there is still no perfect treatment plan, and there are still many shortcomings. Based on this, many researchers are looking for traditional medicine (Hilkens et al., 2024). Ferroptosis is a distinct mechanism that modulates cellular death and is integral to the pathophysiological processes of neurodegenerative disorders and stroke (Wang et al., 2023a). An increasing amount of evidence indicates that ferroptosis is a significant contributor to neurodegenerative disorders and stroke, making pharmacological inhibition of ferroptosis a viable therapeutic target for these conditions (Wang et al., 2023a). The following table provides a list of studies of natural products that are effective anti-ferroptosis in stroke models (Table 3).

**TABLE 3:** Natural products associated with ferroptosis after stroke.

Name	Model	Dose	Influence index/pathway	Ref
Terpenoids				
Ginkgolide B	PC12 OGD/R SD MCAO	10, 20, 40 μM 1,2,4 mg/kg i.p	ACSL4↓, NCOA4↓, FTH1↑, GPX4↑ MDA↓, ACSL4↓, Fe^2+^↓, SOD↑ NCOA4-3 FTH1 interaction	Yang et al. (2024a)
Astragaloside IV	SD MCAO SD SAH SH-SY5Y OGD/R SD MCAO HT-22 Neuro-2a OGD/R C57 MCAO	20 mg/kg i.p 20 mg/kg i.p 40 μg/mL 28 mg/kg i.p 6.25,12.5 and 25 μmol/L 20 mg/kg i.p	ROS↓, MDA↓, GPX4↑, SLC7A11↑ Fe^2+^↓, GSH↑ Nrf2/HO-1 signaling pathway MDA↓, ROS↓, Fe^2+^↓, GPX4↑ SLC7A11↑, GSH↑ Nrf2/HO-1 signaling pathway LDH↓, Fe^2+^↓, MDA↓, ROS↓ GPX4↑ P62/Keap1/Nrf2 pathway LDH↓, MDA↓, GSH↑, Fe^2+^↓ GPX4↑, SLC7A11↑, ACSL4↓ Fto↑, Atf3↑ ACSL4 axis	Zhang et al. (2023a) Liu et al. (2022a) Wang et al. (2023b) Jin et al. (2023)
β-Caryophyllene	Primary astrocytes OGD/R SD, MCAO	10, 20, 40 μM 204,306 and 408 mg/kg i.g	ACSL4↓, COX2↓, 4-HNE↓, MDA↓ Nrf2/HO-1 signaling pathway	Hu et al. (2022)
Ginsenoside Rd	bEnd.3 OGD/R SD MCAO/R	5,10,20 μM 1.5,3,6 mg/mL i.p	ZO-1↑, occludin↑, claudin-5↑ ACSL4↓, COX2↓, GPX4↑, xCT↑ GSH↑, MDA↓, Fe^2+^↓, NRG1↑ PI3K/Akt/mTOR signaling pathway	Hu et al. (2024)
Withaferin A	SH-SY5Y Hemin C57 ICH	10 μM 0.1,1,5 μg/kg i.c.v	SOD↑, GSH-Px ↑, MDA↓, LDH↓, HO-1↑, Nrf2↑ Nrf2/HO-1 signaling pathway	Zhou et al. (2023a)
Rehmannioside A	SH-SY5Y OGD/R SD MCAO	80 μM 80 mg/kg i.p	GPX4↑, SLC7A11↑, SOD↑, MDA↓, GSH↑, ROS↓, MPO↓, GSSG↓ PI3K/AKT/Nrf2 signaling pathway	Fu et al. (2022)
Artesunate	BV2 Haemoglobin SD ICH	5 μM 20,50,70 mg/kg i.p	GPX4↑, ACSL4↓, Fe^2+^↓, ROS↓ AMPK/mTORC1/GPX4 pathway	Xie et al. (2023)
Kellerin	SH-SY5Y OGD/R C57 MCAO	0.1,1,10 μM 10,20 mg/kg i.g	GPX4↑, COX2↓, GSH↑, MDA↓ Fe^2+^↓, Akt/Nrf2 pathway	Mi et al. (2024)
Loureirin C	SH-SY5Y OGD/R C57 MCAO	1,5,10 μM 10,20,40 mg/kg i.g	HO-1↑, GPX4↑, NQO1↑, SOD↑ MDA↓ Keap-1/Nrf2/HO-1 pathway	Liu et al. (2023b)
15,16 Dihydrotansh-inone I	PC12 OGD/R SD MCAO	10 μM 7.5,15,30 mg/kg i.g	GPX4↑, Fe^2+^↓, MDA↓, ROS↓ HO-1↑, Nrf2↑	Wu et al. (2023)
Ecdysterone	PC12 OGD/R SD MCAO	20,40,80 μM 20,40 mg/kg i.p	ROS↓, MDA↓, Fe^2+^↓, ACSL4↓ NCOA4 ↓, FITH↑	Sun et al. (2024a)
Flavonols				
Quercetin	HT-22 OGD/R SD MCAO	30 μM 25,50,70 mg/kg i.g	MDA↓, ROS↓, GSH↑, SOD↑ ACSL4↓, GPX4↑, Fe^2+^↓ Nrf2/HO-1 signaling pathway	Peng et al. (2024)
Baicalein	HT-22 OGD/R C57 tMCAO	0.8,4 μM 10,80 mg/kg i.g	ROS↓ GPX4↑, Fe^2+^↓, ACSL4↓ FTFH1↑, xCT↑, MDA↓ GPX4/ACSL4/ACSL3 pathway	Li et al. (2022)
Baicalin	PC12 Hemin C57 ICH	5,10,20 μM 24 mg/kg i. v	ACSL4↓, GPX4↑, SLC7A11↑ SLC3A2↓, TFRC↑, DMT1↓	Duan et al. (2021)
Calycosin	PC12 OGD/R SD MCAO	15,30,60 μM 5,10,20 mg/kg i.g	Fe^2+^↓, ACSL4↓, GPX4↑, FTFH1↑ TfR1↓ ACSL4-dependent ferroptosis	Liu et al. (2023c)
Vitexin	Primary neurons SD MCAO	0.5, 2.5, 10 nM 50 mg/kg i.p	ROS↓ Keap1/Nrf2/HO-1 signaling pathway	Guo and Shi (2023)
Kaempferol	Primary cortical neurons OGD/R	10 μM	GPX4↑, SLC7A11↑, MDA↓, ROS↓ Fe^2+^↓,4-HNE↓ Nrf2/SLC7A11/GPX4 pathway	Yuan et al. (2021)
Oroxin A	HT22 Hemin C57 SAH	0.5,1,5,10 μM 5 mg/kg i.p	MDA↓, GSH↑, Fe^2+^↓, ROS↓, TNF-α↓, GPX4↑, FTFH1↑, SLC7A11↑ IL-1β↓, IL-6↓, IL-10↑, CD32↓ CD206↑, HO-1↑, FSP1↑	Chen et al. (2024)
Icariside II	Primary astrocytes C57 MCAO	6.25,12.5,25 μM 5,10,20 mg/kg i.p	Fe^2+^↓, GPX4↑ OXPHOS/NF-κB pathway	Gao et al. (2023)
Curcumin	HT22 cells C57 ICH	20 mg/kg p.o	GPX4↑, HMOX1↑, NFE2L2↑	Yang et al. (2021)
(−)-Epicatechin	C57 ICH	5,15,45 mg/kg p.o	ROS↓, Nrf2↑, SOD-1 ↑, AP-1↓ MMP-9↓	Chang et al. (2014)
Carthamin yellow	SD MCAO	20,40 mg/kg p.o	ROS↓, SOD↑, GSH↑, ACSL4↓, TFR1↓ FTH1↑	Guo et al. (2021)
Phenolic				
Rosmarinic acid	C57 (TfR1^−/−^) MCAO	20 mg/kg p.o	GPX4↑, FPN1↑, TfR1↑ ACSL4/LPCAT3/Lox pathway	Jia et al. (2024)
Caffeic acid	SK-N-SH OGD/R SD pMCAO	30 μM 0.4,2,10 mg/kg i.g	ROS↓, SOD↓, GPX↑, MDA ↓ 8-OHdG↓ Nrf2 signaling pathway	Li et al. (2024a)
Salvianolic acid A	Primary cortical neuronal Hemin SD ICH	50 μM 10 mg/kg i.v	GPX4↑, ROS↓, xCT↑ Akt/GSK-3β/Nrf2 pathway	Shi et al. (2024)
Paeonol	HT22 cells Hemin C57 ICH	5,10,20,40 μM 10,20,30 mg/kg i.p	GSH↑, MDA↓, ROS↓, SLC7A11↑ ACSL4↓ HOTAIR/UPF1/ACSL4 pathway	Jin et al. (2021)
Carvacrol	Gerbils mice MCAO	25,50,100 mg/kg p.o	GSH↑, MDA↓, ROS↓, Fe^2+^↓ TFR-1↓, FPN1↑	Guan et al. (2019)
Resveratrol	Primary neurons SD MCAO	5,10,15,20 μM 30 mg/kg i.p	GSH↑, Fe^2+^↓, ACSL4↓, GPX4↑ ROS↓	Zhu et al. (2022a)
Rhein	HT22 OGD/R SD MCAO	10,20,40 μM 20,50,100 mg/kg i.p	Fe^2+^↓, ROS↓, GPX4↑, GSH↑ NRF2/SLC7A11/GPX4 pathway	Liu et al. (2023d)
Alkaloids				
Berberine	C57 MCAO	Fecal microbiota transplantation	GSH↑, MDA↓, SLC7A11↑, GPX1↑ ACSL4↓	Wang et al. (2023c)
Dauricine	SH-SY5Y RSL3 C57 ICH	0.03 μM–3 μM 5 mg/kg i.g	GPX4↑, GSR↑, ROS↓, MDA↓ Fe^2+^↓	Peng et al. (2022)
Traditional Chinese medicine formulations				
Angong Niuhuang Wan	SD MCAO SD ICH	257,514 mg/kg p.o	LPO↓, Fe^2+^↓, GPX4↑ PPARγ/AKT/GPX4 pathway	Bai et al. (2024)
DiHuang YinZi	SD MCAO	0.2,0.4,0.8 mg/kg p.o	MDA↓, ROS↓, GSH↑, Fe^2+^↓, GPX4↑ P53/SLC7A11 pathway	Yang et al. (2024b)
Naodesheng Pills	SH-SY5Y OGD/R SD MCAO	3.125, 6.25, 12.5, 25 μg 0.54,1.08 mg/kg p.o	TFR1↓, GPX4↑, DMT1↓, GSH↑ MDA↓, SOD↓ ERK1/2 Pathway	Yang et al. (2024c)
Naotaifang	BV2 OGD/R SD MCAO SD MCAO	9,18,27 g/kg 9,18,36 mg/kg p.o 27 g/kg p.o	Fe^2+^↓, ROS↓, GPX4↑, MDA↓ BMP6/SMADs Pathway Fe^2+^↓, ROS↓, GPX4↑, MDA↓ TFR1↑, GSH↑, DMT1↓, SLC7A11↑	Liao et al. (2023) Lan et al. (2020)
Tongluo Decoction	HUVECs OGD/R SD MCAO PC12 OGD/R SD MCAO	24 g/kg 6,12,24 mg/kg p.o 6,24 mg/kg p.o	ROS↓GPX4↑, MDA↓, Fe^2+^↓ Nrf2/ARE/SLC7A11 Pathway ROS↓, SOD↑, MDA↓, GPX4↑ ACSL4↓	Li et al. (2024b) Hui et al. (2022)
Xingnaojing injection	SH-SY5Y OGD/R SD MCAO	0.25%,0.5%, 1% 0.18 mL/100g i.p	GPX4↑, FPN↑, HO-1↑, COX-2↓, TFR ↓, DMT1↓	Liu et al. (2023e)
Tongqiao Huoxue Decoction	PC12 OGD/R SD MCAO	10,20,40 μM 3,6,12 g/kg i.p	SOD↑, ROS↓, MDA↓, GPX4↑ FTH1↑, ACSL4↓	Ou et al. (2024)
Salvia miltiorrhiza	C57 MCAO	200 mg/kg p.o	4-HNE↓, MDA↓, FPN1↓, GPX4↓ ACSL4↓	Ko et al. (2023)
Paeoniae Radix Rubra extract	HT22 OGD/R SD MCAO	100 μg/mL 0.27,0.54,1.08 g/kg p.o	GPX4↑, FTH1↑, Beclin1↑, ROS↓ LC3 II↑, MDA↓, GSH↑	Zhao et al. (2023b)
Danlou tablet	hy926 cell OGD/R C57 MCAO	1400,2800 mg/kg p.o	GPX4↑, COX2↑, SLC7A11↑	Liu et al. (2024)
Danhong Injection	HT22 OGD/R C57 MCAO	5,10,20 μL/mL 2.5,5 mL/kg i.p	SOD↑, MDA↓, GSH↑ SATB1/SLC7A11/HO-1 pathway	Zhan et al. (2023)
Salvia miltiorrhiza Bge. (process with porcine cardiac blood)	SH-SY5Y OGD/R SD MCAO	2.5,5,10 μg/mL 3,6,12 g/kg p.o	GSH↑, MDA↓, SOD↑, ROS↓, GLRX5↓ SLC7A11/GPX4 pathway	Zhou et al. (2024a)
Roots of *Astragalus propinquus* schischkin	Granule Injection SD MCAO	2.5 g/kg p.o 2 mL/kg i.p	HO-1↑, Nrf2↑, GPX4↓, XCT↑ SLC3A2↑, FPN1↑, TRPC6↓ DMT1↓, TFR↓	Chen et al. (2022a)
Polysaccharide				
Neutral polysaccharide from Gastrodia elata	HT22 OGD/R C57 MCAO	100,250,500 μg 0.5,1,2 mg/kg	MDA↓, ROS↓, GSH↑, Fe^2+^↓ GPX4↓, SLC7A11↑ NRF2/HO-1 pathway	Zhang et al. (2024)
Polysaccharide from Salvia miltiorrhiza Bunge	PC12 OGD/R	20,40,60 μg/mL	ROS↓, Fe^2+^↓, SLC7A11↑, GSH↑ GPX4↓, MDA↓ Nrf2/HO-1 pathway	Meng et al. (2022)
Other Compounds				
Cottonseed oil	SD MCAO	1.3 mL/kg	GPX4↑, XCT↑, HO-1↑, FTH1↑, SOD↑, ACSL4↓, GSH↑, MDA↓	Sun et al. (2023)
Mastoparan M	HT22 OGD/R C57 MCAO	17, 34, 68 nM 20,40,80 μg/kg i.p	ROS↓, Fe^2+^↓, Nrf2↑, XCT↑, GPX4↓ NRF2-GPX4 pathway	Wang et al. (2024a)
Melatonin	HT22 OGD/R C57 MCAO HT22 OGD/R C57 MCAO	20,40 μM 5,10 mg/kg i.p 20,40 μM 5,10 mg/kg i.p	ROS↓, GSH↑ ACSL4/CYP1B1 pathway ROS↓, ACSL4↓, MDA↓, SOD↑ MDM2↑, GSH↑	Sun et al. (2024b) Ji et al. (2024)
Dl-3-n-butylphthalide	SD MCAO	10,20,30 mg/kg i.p	SOD↑, ROS↓ MDA↓, TFRC↓, GSH↑, GPX4↑, SLC7A11↑	Xu et al. (2023b)
Taurine	HT22 Hemin C57 SAH	20 μM 400 mg/kg i.p	MDA↓, ROS↓, SOD↑, GPX4↑ SLC7A11↑, Fe^2+^↓ GABAB/AKT/GSK3β/β-catenin	Liu et al. (2022b)
Crocin	C57 ICH	40 mg/kg p.o	GSH-px↑, SOD↑, MDA↓, Nrf2↑ GPX4↑, FTH1↑, SLC7A11↑	Wang et al. (2022b)

### 5.1 Terpenoids

The structure of terpene compounds is built from isoprene units (C5 units), which are related to methylglutaric acid. These oxygen-containing derivatives can be alcohol, aldehydes, ketones, carboxylic acids, esters, among others. Terpenes are widely found in nature and are the main plant metabolites of essence, resin, pigment, among others, which constitute some plants. Terpenoids also include hormones and vitamins from animals. Recently, many scholars have found that terpenoids have anti-ferroptosis effects.

**Ginkgolide B** (PubChem CID: 65243) is a terpenoid compound that is generated from plants, namely, from *Ginkgo biloba L*. It exhibits a range of pharmacological effects, including inhibiting platelet aggregation, reducing inflammation, acting as an antioxidant, and scavenging free radicals (Chen et al., 2022b). Ginkgolide B inhibits the overproduction of ROS, MDA, ACSL4, NCOA4, and Fe^2+^, while simultaneously increasing the activities of antioxidative enzymes GPX4 and SOD, and FITH1 (Yang et al., 2024a). It achieves its anti-ferroptosis effect by disrupting the NCOA4-FTH1 interaction (Yang et al., 2024a). In addition, molecular docking, and microscale thermophoresis assay were conducted to explore the combination of Ginkgolide B and NCOA4. Therefore, ginkgolide B may be a candidate for the treatment of ferroptosis after stroke.

**Astragaloside IV** (PubChem CID:13943297) is a lanolin alcohol-derived tetracyclic triterpene saponin extracted from *Astragalus membranaceus*. It is a white powder with the molecular formula C_41_H_68_O_14_. Studies indicate that astragaloside IV exerts a neuroprotective effect through multiple mechanisms, including its anti-inflammatory, anti-oxidative, and anti-apoptotic properties that safeguard nerve cells (Yao et al., 2023). Additionally, it regulates nerve growth factor, inhibits neurodegeneration, and facilitates neuron regeneration (Yao et al., 2023). In MCAO rats, astragaloside IV can inhibit the expression of inflammatory factors TNF-α, IL-1β, IL-6, and NF-κB, increasing the levels of SLC7A11 and GPX4 (Zhang et al., 2023a). Mechanism studies indicate that astragaloside IV triggered the Nrf2/HO-1 signaling pathway and alleviated ferroptosis due to ischemic stroke induction (Zhang et al., 2023a). Notably, ML385 inhibited these effects, and astragaloside IV increased P62 and Nrf2 levels while decreasing Keap1 levels. P62 silencing decreased astragaloside IV’s effects on the P62/Keap1/Nrf2 pathway and ferroptosis (Wang et al., 2023b). Another study showed that astragaloside IV promoted the transcription of Fto by regulating Atf3, resulting in a decrease of Acsl4 levels, thus improving neuronal injury in ischemic stroke by inhibiting ferroptosis (Jin et al., 2023). Interestingly, in the SAH model, astragaloside IV also exerts its anti-ferroptosis effect through the Nrf2/HO-1 pathway (Liu et al., 2022a). Although many studies have shown that it can treat central nervous system (CNS) diseases, previous pharmacokinetic studies have shown that AS-IV has a poor oral bioavailability and membrane permeability (Li et al., 2023b). There are also studies that suggest that it may affect CNS diseases through intestinal bacteria (Li et al., 2023b). If it is to be developed as a drug, further mechanism studies are needed.

**β-Caryophyllene** (PubChem CID: 26318) is a class of bicyclic sesquiterpenes found in lemon, grapefruit, nutmeg, pepper, raspberry, blackcurrant, cinnamon leaf oil, and clover leaf oil, whose molecular formula is C_15_H_24_ (Sharma et al., 2016). The *in vivo* study demonstrated that BCP enhanced neurological scores, infarct volume, and pathological features following MCAO/R. They found that BCP significantly increased the nuclear translocation of Nrf2 and activated the Nrf2/HO-1 pathway, thereby safeguarding against ferroptosis (Hu et al., 2022). β-Caryophyllene decreased OGD/R-induced ROS generation and iron accumulation (Hu et al., 2022). Furthermore, the neuroprotective effects of β-Caryophyllene were reversed by the Nrf2 inhibitor ML385. β-Caryophyllene is a food additive that has been cleared by the Food and Drug Administration and is generally thought to be safe. β-Caryophyllene is also highly available orally and has been studied in RCT in vascular diseases, which I think is a good candidate for the treatment of stroke (Yamada et al., 2023).

**Ginsenoside Rd** (PubChem CID: 11679800) exhibits diverse pharmacological properties including cardiovascular protection, neuroprotection, anti-aging, anti-tumor, and more (Chen et al., 2022c). The chemical formula of the compound is C_48_H_82_O_18_. Ginsenoside Rd could increase the expression of ZO-1, occluding-1, and claudin-5 in cerebral micro vessels and bEnd.3 cells on the same side of the brain, leading to a decrease in the loss of endothelial cells and leakage of Evans blue dye. As a result, ginsenoside Rd eventually enhances the integrity of the BBB following cerebral I/R injury (Hu et al., 2024). Ginsenoside Rd can mitigate the breakdown of the BBB generated by ischemic stroke by reducing ferroptosis in endothelial cells (Hu et al., 2024). Functionally, ginsenoside Rd protected against tight junction loss and leakage of the BBB by increasing the expression of NRG1, which in turn activated the tyrosine kinase ErbB4 receptor. This activation subsequently triggered the PI3K/Akt/mTOR signaling pathway, ultimately preventing ischemic stroke-induced ferroptosis in endothelial cells (Hu et al., 2024). Despite its potent pharmacological efficacy, ginsenoside Rd is present in plants in relatively low quantities, making its production from ginseng an expensive process.

**Withaferin A** (PubChem CID: 265237) is a steroid ester plant metabolite with a molecular formula of C_28_H_38_O_6_. Withaferin A treatment was found to suppress ferroptosis and reduce oxidative stress (OS)-related damage in both an *in vivo* model and an *in vitro* model of ICH (Zhou et al., 2023a). These effects were attributed, at least in part, to the augmented translocation of Nrf2 and increased expression of HO-1 (Zhou et al., 2023a). More precisely, withaferin A can enhance the production of HO-1, SOD, GSH-Px, and Nrf2 while suppressing the production of MDA (Zhou et al., 2023a). However, the sample size of this study is too small, so more pharmacodynamics studies should be carried out in the future, and then mechanism studies should be carried out.

**Rehmanioside A** (PubChem CID: 6325881) is an iridoid glycoside that promotes immunity, replenishes blood, and lowers blood sugar (Liu et al., 2017). The chemical formula is C_21_H_32_O_15_. Furthermore, current research has shown that rehmannioside A could decrease OS, enhance cognitive function, and provide a safeguarding impact on neurons (Sun et al., 2019a). Rehmannioside A exhibits neuroprotective properties and enhances cognitive function following cerebral ischemia by suppressing ferroptosis and activating the PI3K/AKT/Nrf2 and SLC7A11/GPX4 signaling pathways (Fu et al., 2022). Specifically, the rehmannioside A group significantly improved the cognitive impairment and neurological deficits compared to the model group and reduced cerebral infarction in MCAO rats (Fu et al., 2022). Furthermore, the rehmannioside A group demonstrated a noticeable increase in cell viability and a reduction in H_2_O_2_-induced toxicity (Fu et al., 2022). Further research revealed a significant increase in the expression of p-PI3K, p-Akt, nuclear Nrf2, HO-1, and SLC7A11 in the rehmannioside A group compared to the model group (Fu et al., 2022). In addition, rehmannioside A inhibits the release of pro-inflammatory mediators from microglia and promotes M2 polarization *in vitro*, thereby protecting co-cultured neurons from apoptosis by inhibiting NF-κB and MAPK signaling pathways (Xiao et al., 2021).

**Artesunate** (PubChem CID: 6917864) possesses the attributes of cost-effectiveness, rapid onset, little toxicity, and resistance development challenges. Recent research has discovered that artesunate could trigger ferroptosis without relying on xCT (Sun et al., 2019b). Chao Qin’s research found that artesunate caused LPO and the formation of ROS in BV2 cells stimulated by LPS (Xie et al., 2023). However, the use of the ferroptosis inhibitor Fer-1 effectively restored these effects (Xie et al., 2023). Furthermore, artesunate induced ferroptosis in ICH M1-polarised BV2 cells primarily through the AMPK/mTOR/GPX4 axis and partially through Akt phosphorylation inhibition, but not by severing the link between mTORC1 and lysosomes (Xie et al., 2023). However, the *in vitro* data cannot be considered pharmacological or clinically relevant. More animal experiments should be carried out in the future to verify the anti-ferroptosis effect of artesunate.

**15,16-Dihydrotanshinone I** (PubChem CID: 11425923, DHT), a lipophilic tanshinone with the chemical formula C_18_H_14_O_3_, is isolated from the root of *Salvia miltiorrhiza Bunge* [Lamiaceae; Salviae miltiorrhizae radix et rhizoma]. The *in vitro* investigation on ferroptosis was reduced by DHT, as evidenced by a decrease in lipid ROS generation, an increase in GPX4 expression and the ratio of GSH/GSSG, and an improvement in mitochondrial function (Wu et al., 2023). The inhibitory effect of DHT on ferroptosis was decreased after Nrf2 silencing. Compared with the pMACO group, DHT significantly increased the expression of GPX4 and reduced the GSH-Px activity. The inhibitory effect of DHT on ferroptosis and its underlying mechanism in pMCAO rats were examined *in vivo*. However, relying on a few indicators of ferroptosis to prove the anti-ferroptosis effect of DHT is far from sufficient. More in-depth mechanism research is needed in the future.

**Kellerin** (PubChem CID: 40580807) inhibits microglial activation, thereby exerting a potent anti-inflammatory effect. Its molecular formula is C_32_H_26_O_12_. Kellerin inhibited the production of mitochondrial ROS *in vitro*, thereby improving the neuronal injury caused by OGD/R and suppressing ferroptosis (Mi et al., 2024). Kellerin directly contacted Akt and raised its phosphorylation, which resulted in an increase in Nrf2 nuclear translocation and the production of its downstream antioxidant genes (Mi et al., 2024). Kellerin provided protection against IS and prevented ferroptosis *in vivo*, evidenced by its ability to enhance the expression of GSH and GPX4 while decreasing MDA and Fe^2+^ levels.

Additionally, molecular docking, in conjunction with drug affinity responsive target stability assay (DARTS) and cellular thermal shift assay (CETSA), was conducted to evaluate the possible target proteins for kellerin. The findings indicate that kellerin significantly raised the level of Akt phosphorylation, indicating that it can bind to Akt and encourage phosphorylation. In conclusion, we assert that kellerin may serve as a candidate therapeutic agent for the ongoing management of ferroptosis in stroke.

**Loureirin C** (PubChem CID: 14157896) is a type of dihydrochalcone obtained from resin extracted from the stem of *Chinese Dragon’s Blood* (Dracaena cochinchinensis S.C. Chen), with the molecular formula C_16_H_16_O_4_. In mice following MCAO/R, loureirin C not only significantly reduced brain damage and prevented neurons from ferroptosis, but it also reduced ROS accumulation in ferroptosis in a dose-dependent manner following OGD/R (Mi et al., 2024). In addition, loureirin C raises the amount of NQO1, GPX4, and HO-1 following ischemic stroke (Mi et al., 2024). To further prove the anti-ferroptosis effect of Loureirin C, they employed ferroptosis activator (Erastin), aptoptosis inhibitors and pyroptosis inhibitors (Z-VAD-FMK) in OGD/R induced SH-SY5Y cells. The findings suggested Loureirin C could increase the cell survival rate in OGD/R cell model in the presence of Z-VAD-FMK (10 μM) with or without erastin (10 μM). Co-Immunoprecipitation assay suggested that Loureirin C could reduce the level of Nrf2 binding to Keap1. All things considered, we think loureirin C is a candidate medication acting as an antioxidant in ferroptosis stroke treatment.

**Ecdysterone** (PubChem CID: 5459840), found in *Achyranthes bidentata Blume*, is a crucial active plant metabolite that has enhanced its medical potential by virtue of its antioxidant and neuroprotective properties. The chemical formula of the compound is C_27_H_44_O_7_. In MCAO rats, ecdysterone improves ischemic stroke by inhibiting ferroptosis and OS, resulting from increased GSH levels and decreased MDA, ROS, LPO, and Fe^2+^ levels (Sun et al., 2024a). Further research has demonstrated that ecdysterone inhibits ferroptosis in MCAO rats via ACSL4/NCOA4/FTH1pathway (Sun et al., 2024a). To verify the binding between ecdysterone and ACSL4, CETSA was carried out. Ecdysterone enhanced the thermal stability of ACSL4, suggesting that ecdysterone directly interacts with ACSL4 within the cellular environment. This data indicates that ACSL4 is a direct target of ecdysterone. Nevertheless, there is a paucity of research regarding stroke in relation to ecdysterone. Consequently, we assert that this is not a viable candidate drug. Future investigations of the efficacy of mechanisms are essential.

### 5.2 Flavonoids

Flavonoids were originally referred to as a class of compounds derived from the backbone of 2-phenylchromenone. It now refers to a series of compounds formed by two benzene rings connected to each other through three carbon atoms, i.e., a general term for a class of compounds with a C6-C3-C6 structure. Plants widely distribute flavonoids, most of which exist as glycosides or carbon glycosides produced by sugar synthesis, while others exist in their free form. Flavonoids, particularly quercetin, have demonstrated significant anti-ferroptosis effects *in vitro* and *in vivo* (Wendlocha et al., 2024). The favorable effects of this group of chemicals are mainly attributed to their antiatherogenic, antithrombotic, and antioxidant properties (Wendlocha et al., 2024).

**Quercetin** (PubChem CID: 5280343), a prevalent flavonoid present in medicinal plants, demonstrates therapeutic properties against a range of illnesses, such as alcoholic hepatitis, renal IR injury, and cancer (Chen et al., 2021c). Pharmacokinetic investigations have shown that quercetin can penetrate the BBB, indicating its potential to protect against neurodegenerative diseases (Pavlović et al., 2023; Javadinia et al., 2022). Quercetin enhances neurological function, reduces the size of brain tissue damage caused by MCAO in rats, and mitigates pathological characteristics (Peng et al., 2024). Additionally, quercetin promotes the survival of HT-22 cells when exposed to H_2_O_2_ and erastin (Peng et al., 2024). Specifically, quercetin can inhibit MDA, ROS, and Fe^2+^ expression while increasing SOD and GSH expression (Peng et al., 2024). It was discovered that quercetin inhibited ferroptosis both *in vitro* and *in vivo* by up-regulating GPX4 and FTH1 and down-regulating ACSL4 (Peng et al., 2024). Previous detailed publications on quercetin’s research with stroke suggest that it may be a viable candidate for stroke treatment (Zhang et al., 2022b).

**Baicalein** (PubChem CID: 5281605) is known for its various pharmacological properties, including antibacterial, antiviral, anti-inflammatory, antioxidant, and anti-tumor effects (Srivastava et al., 2021). Pharmacokinetic investigations have demonstrated that baicalein can cross BBB and spread throughout the cerebral nuclei (Zhu et al., 2012). Because of its low toxicity and therapeutic properties, baicalein, a natural bioactive molecule, has been extensively studied for its potential in treating stroke. Baicalein reduces iron levels, LPO generation, and morphological characteristics associated with ferroptosis in the brain tissues of MCAO mice (Li et al., 2022). Furthermore, baicalein suppressed ferroptosis by modulating the expression levels of GPX4, ACSL4, and ACSL3 in OGD/R cells, MCAO mice, and RSL3-stimulated HT22 cells (Li et al., 2022). A preliminary study of baicalin in the preparation of astragali decoction conducted by Zheng et al. reported that after oral administration to rats, baicalin was almost undetectable in serum because *Escherichia coli* hydrolyzed baicalin to baicalein and oroxylin A (Jung et al., 2012). In contrast, a large amount of metabolic jaundice was detected in the gut contents and a small amount of baicalin was detected in the blood of sterile rats 2 h after oral baicalin administration at the same dose. In normal rats, baicalin but not baicalein was detected in the blood soon after oral administration. This indicates that baicalin was not directly absorbed into the blood and that only its transformation into baicalein by the gut microbiota allowed it to enter the blood circulation.

**Baicalin** (PubChem CID: 64982) is a flavonoid compound with the molecular formula C_21_H_18_O_11_. It has pharmacological characteristics, including antioxidant, antiapoptotic, and neuroprotective actions, in several illnesses (Zhou et al., 2019; Chen et al., 2018). Baicalin also can inhibit the development of ferroptosis in ICH (Duan et al., 2021). It has been reported to exhibit neuroprotective effects against ICH-induced brain injury as well as reduce iron deposition in multiple tissues (Duan et al., 2021). Baicalin enhanced cell viability and suppressed ferroptosis in rat pheochromocytoma PC12 cells treated with hemin, erastin and RSL3 (Duan et al., 2021). Specifically, baicalin can promote the expression of GPX4 and SLC7A while inhibiting SLC3A2 and DMT1 (Duan et al., 2021). However, this experiment only detected a few indicators of ferroptosis and did not perform more in-depth research on the mechanisms involved. More and deeper studies are needed in the future.

**Calycosin** (PubChem CID: 5280448) is a typical phytoestrogen and possesses a variety of pharmacological activities, including anticancer, anti-cardiotoxicity, anti-diabetic nephropathy, and anti-cerebral ischemic activities, its molecular formula is C_16_H_12_O_5_ (Deng et al., 2021). The administration of calycosin resulted in a reduction in ferroptosis, as shown by the measurement of iron accumulation, MDA, SOD, ceramide, and ROS levels, as well as the expression of ferroptosis-related proteins (ACSL4, TfR1, FTH1, and GPX4) (Liu et al., 2023c). However, the sample size is too small, and more basic research should be needed in the future.

**Vitexin** (PubChem CID: 5280441) is a biologically active flavonoid chemical obtained from many culinary and medicinal plants. The chemical formula of the compound is C_21_H_20_O_10_. Vitexin reduced ferroptosis and protects brain tissue through the Keap1/Nrf2/HO-1 pathway (Guo and Shi, 2023). In comparison to the Model group, treatment with Vitexin (0.5, 2.5, and 10 nM) significantly reduced the levels of HO-1, SLC7A11, and GPX-4, while increasing the levels of Keap1 and Tfr1. However, the oral bioavailability of vitexin is low and high doses of vitexin may lead to elevated liver enzymes, so we do not consider it a very good candidate drug.

**Kaempferol** (PubChem CID: 5280863), with a chemical formula of C_15_H_10_O_6_, is a prominent bioflavonoid that can be found in a variety of fruits, vegetables, and medicinal plants. Kaempferol exhibits neuroprotective, antioxidant, and anti-cancer attributes against several disorders linked to lipid oxidation, such as stroke, Alzheimer’s disease, and cancer (Nezhad Salari et al., 2024). Kaempferol increased SLC7A11, GPX4, and Nrf2 in OGD/R-treated neurons (Yuan et al., 2021). Kaempferol can also alleviate the accumulation of Fe^2+^ in OGD/R cells (Yuan et al., 2021). Kaempferol offers defiance against OGD/R-induced ferroptosis by activating the Nrf2/SLC7A11/GPX4 signaling pathway, at least to some extent (Yuan et al., 2021). Nonetheless, the entire article relies on data derived from *in vitro* experiments, which are inadequate for evaluating pharmacological effects if it is a pan-assay interfering drug. Animal experiments should be carried out in the future to fully verify its pharmacological effects.

**Oroxin A** (PubChem CID: 5320313), also known as baicalein-7-O-glucoside, is a highly effective flavonoid with the molecular formula C_21_H_20_O_10_. Oroxin A against ferroptosis after SAH *in vivo* (Chen et al., 2024). Following SAH, the levels of FTH1, GPX4, and SLC7A11 exhibited a decline, which was subsequently reversed by oroxin A treatment (Chen et al., 2024). Oroxin A can control ferroptosis through the Nrf2/GPX4 pathway and the CoQ10-FSP1 pathway (Chen et al., 2024). The neuroprotective properties of oroxin A are mediated through the Nrf2/GPX4 pathway, and the inhibitory effects of Oroxin A on ferroptosis and neuroinflammation depend on the transcriptional response of Nrf2. However, oroxin A is poorly utilized when taken orally, and it cannot cross the BBB. We consider it not a promising candidate drug.

**Icariside II** (PubChem CID: 5488822), a naturally occurring flavonoid companies derived from traditional Chinese medicinal *Epimedium sagittatum* (Siebold & Zucc.) Maxim. Its molecular formula is C_27_H_30_O_10_, and it possesses several pharmacological activities, such as antioxidant stress, anti-neuroinflammatory, anti-osteoporotic, and anti-cancer properties (Zhang et al., 2023b). Icariside II preconditioning exerts neuroprotective effects by activating the astrocytic Nrf2-mediated OXPHOS/NF-κB/ferroptosis axis (Gao et al., 2023). To be more specific, icariside II preconditioning greatly lowered the production of ROS, MDA, GPX4 levels, SOD2 activity, and SIRT5 activity in MCAO mice (Gao et al., 2023). Compared with the MCAO group, the icariside II precoding group increased the expression of HO-1, NQO-1, SIRT5, and GPX4 (Gao et al., 2023). Surface plasmon resonance assay investigates demonstrate that ICS II interacts with the Nrf2 protein, exhibiting an equilibrium constant (K_D_) of 1.033 × 10^−4^ M. Furthermore, Electrophoretic mobility shift assay confirmed that ICS II preconditioning enhanced the binding of Nrf2 to ARE at 2, 4, and 24 h. A competitive assay verified that the cold probe impeded the DNA-binding activity of Nrf2, hence further substantiating the specificity of the binding. All of these results suggest that ICS II probably activates a Nrf2–ARE signaling pathway by targeting Nrf2. LC-MS analysis demonstrated that the quantities of ICS II in the brain homogenates of mice following MCAO injury were elevated compared to those in the sham group, indicating that ICS II can cross the BBB. Therefore, we state that Icariside II is a viable pharmacological candidate for stroke treatment.

**Curcumin** (PubChem CID: 969516) is a commonly found phenolic molecule derived from the rhizome of *Curcuma longa L*. with a chemical formula of C_21_H_20_O_6_. It is known to possess strong antioxidants, anti-inflammatory, and neuroprotective properties when used in pharmaceutical applications (Zhang et al., 2018). Curcumin has been observed to have the capacity to modulate ferroptosis processes associated with cancer, tissue injury, and other diseases, as evidenced by the *in vitro* and *in vivo* findings described above (Foroutan et al., 2024). Nevertheless, the effectiveness of curcumin is significantly hindered by its undesired water solubility, inadequate oral bioavailability, inefficiency in crossing the BBB, and other physiological barriers (Tran and Tran, 2020). In the experiments of Cong Yang et al., they encapsulated curcumin in Polymer-based nanoparticles (Cur-NPS) and explored the effect of these Cur-NPs to enhance Cur delivery both *in vitro* and *in vivo* (Yang et al., 2021). The findings demonstrated that Cur-NPs effectively inhibited erastin-induced ferroptosis in HT22 murine hippocampus cells (Yang et al., 2021). However, all the indicators related to ferroptosis are cell experiments. More animal experiments should be carried out in the future. Moreover, due to the characteristics of curcumin, we believe that curcumin cannot be a candidate drug for the treatment of ICH.

**(−)-Epicatechin** (PubChem CID: 72276) is a naturally derived flavanol molecule, represented by the molecular formula C_15_H_14_O_6_, which is abundantly present in tea, chocolate, and many botanical drugs. The molecule has a crucial antioxidant role by binding phenolic hydroxyl groups and free radicals, hence achieving free radical scavenging activities. (−)-Epicatechin was found to have the ability to decrease ferroptosis *in vivo*, as evidenced by its reversal of the rising levels of ROS and Fe^2+^ (Chang et al., 2014). Furthermore, the levels of lipid ROS and LC3 in H9C2 cells were reduced with (−)-epicatechin treatment (Chang et al., 2014). Additionally, the processes of autophagy and ferroptosis were also mitigated in a manner that depended on the dosage, as shown *in vitro* (Chang et al., 2014). The co-cultivation of the USP14 inhibitor IU1 and (−)-epicatechin demonstrated that (−)-epicatechin controls ferroptosis via influencing the USP14-autophagy pathway (Chang et al., 2014). However, quantitative experimental methods detected the expressions of several ferroptosis-related genes, including SOD, NQO1, and MDA; these methods are inadequate for evaluating pharmacological effects if the drug is a pan-assay interfering drug.

**Carthamin yellow** (PubChem CID: 12305280) is derived from the *Carthamus tinctorius L*., and its chemical formula is C_21_H_22_O_11_. Carthamin yellow mitigated the effects of MCAO-induced ferroptosis by reducing iron and ROS accumulation, decreasing LPO, and restoring the expression levels of proteins linked to ferroptosis (Guo et al., 2021). In addition, carthamin yellow therapy suppressed the accumulation of Fe^2+^ and ROS and restored the levels of ACSL4, TFR1, GPX4, and FTH1 proteins in the brain (Guo et al., 2021). Compared with the model group, carthamin yellow group also increased SOD and GSH and decreased MDA levels (Guo et al., 2021). Carthamin yellow can cross the BBB; however, its oral bioavailability is limited, and the precise target mechanisms remain ambiguous. Further comprehensive research on the mechanism is necessary for its potential development into medication in the future.

### 5.3 Polyphenols

Polyphenols are formed by direct connection between hydroxyl (-OH) groups and aromatic nuclei (benzene rings or condensed benzene rings). Polyphenols are widely present in nature and can be classified into volatile phenols and non-volatile phenols based on their volatility. Polyphenols were long believed to be primarily anti-nutrients and not particularly essential to human nutrition (Rana et al., 2022). However, recent research studies have now shown that polyphenolic substances do many biologically important things, including protecting against metabolic disorders and chronic diseases and acting as an antioxidant (Ganesan and Xu, 2017). Consuming polyphenol-rich foods has been associated with a range of health benefits, including optimizing cardiometabolic health and to a lesser extent positively impacting brain functioning in humans.

**Rosmarinic acid** (PubChem CID: 5281792) is a water-soluble compound with the chemical formula C_18_H_16_O_8_. Rosmarinic acid is a naturally occurring water-soluble phenolic acid compound characterized by its unstable properties, low lipid solubility, and limited cell membrane permeability (Noor et al., 2022). To overcome this drawback, Cui ling Jia et al. used rosmarinic acid encapsulated in liposomes (RosA-LIP) (Jia et al., 2024). The administration of RosA-LIP improved the structural defects of mitochondria and reinstated the integrity of mitochondrial cristae (Jia et al., 2024). The RosA-LIP treatment resulted in an increase in the activity of SOD and CAT, as well as the levels of Nrf2, HO-1, and GSH (Jia et al., 2024). Compared with the model group, the RosA-LIP group significantly reduced the expression of Ptgs2, ACSL4, LPCAT3, 12-Lox, MDA, and 4-HNE (Jia et al., 2024). In addition, RosA-LIP has shown the capability to specifically inhibit the expression of TfR1 in BMECs, resulting in a decrease in the uptake of iron in the brain and minimizing the occurrence of ferroptosis, which is dependent on ACSL4/LPCAT3/Lox, in the ischemic brain (Jia et al., 2024). The specific target of rosmarinic acid remains undetermined, necessitating future investigation for accurate identification.

**Caffeic acid** (PubChem CID: 689043) is a polyphenolic compound present in a vast array of dietary plant metabolites. Its molecular formula is C_9_H_8_O_4_. In MCAO rat brain and in OGD/R-treated SK-N-SH cells *in vitro*, caffeic acid decreased the expression of TFR1 and ACSL4, and increased the synthesis of glutathione via the Nrf2 signaling pathway to inhibit ferroptosis (Li et al., 2024a). Application of ML385, an Nrf2 inhibitor, blocked the neuroprotective effects of caffeic acid in both *vivo* and *in vitro* models, evidenced by excessive accumulation of iron ions and inactivation of the ferroptosis defense system. This is the initial study on the investigation of caffeic acid in relation to stroke. We suggest that more research should be conducted in the future to validate the efficacy of Caffeic acid on stroke.

**Salvianolic acid A** (PubChem CID: 5281793) has been identified as a particularly effective agent that possesses anti-inflammatory and antioxidant properties, as well as the ability to modulate the integrity and functionality of the BBB (Liu et al., 2021). Salvianolic acid A reduced MDA and Fe^2+^ production and reversed the downregulation of GSH, XCT, and GPX4 (Shi et al., 2024). Further research shows that salvianolic acid A inhibits ferroptosis after ICH through Akt/GSK-3β/Nrf2 signaling pathway (Shi et al., 2024). A pharmacokinetic investigation demonstrated that circulatory system exposure to Salvianolic acid A was comparable between sham controls and I/R rats; however, brain exposure to Salvianolic acid A was markedly elevated in I/R rats compared to sham controls (fold change of 9.17), indicating that the increased exposure to Salvianolic acid A facilitated its neuroprotective effect (Feng et al., 2017). In conclusion, we conclude that Salvianolic A is a potential therapeutic agent for stroke treatment.

**Carvacrol** (PubChem CID: 10364) is a natural compound that occurs in the leaves of several plants and botanical drug including wild bergamot, thyme and pepperwort, but which is most abundant in *Origanum vulgare L*. The molecular formula of carvacrol is C_10_H_14_O. Compare with the model group, carvacrol could increase SOD, GSH-Px, and CAT in MCAO mice and reduce the expression of MDA and Fe^2+^ (Guan et al., 2019). *In vitro* experiments indicated that carvacrol significantly decreased the levels of MDA, H2AX protein expression, and hippocampal neuron impairment compared to those in the anoxia/reoxygenation group, but these effects were reversed by silencing GPx4. This study performed *in vivo* and *in vitro* experiments to illustrate the protective impact of carvacrol on stroke; nonetheless, there is an insufficient amount of research on carvacrol in the context of stroke, necessitating further pharmacological and mechanistic investigations in the future.

**Resveratrol** (PubChem CID: 445154), a nonflavonoid polyphenol molecule, possesses potent anti-inflammatory, anti-OS, and anti-apoptotic properties. Pretreatment with resveratrol can effectively prevent iron overload, enhance neuronal survival, increase levels of GSH, reduce levels of ROS, decrease the expression of ACSL4 protein, increase the expression of Ferritin and GPX4 proteins, and mitigate damage to mitochondrial structure following OGD/R injury *in vitro* (Zhu et al., 2022a). Additionally, resveratrol pretreatment may improve the rate of neuronal survival *in vitro* and lessen the effects of erastin and RSL3-induced ferroptosis (Zhu et al., 2022a). Resveratrol has been the subject of extensive research on stroke, but the bioavailability is only 1%. Future research should address this drawback. To sum up, we believe that resveratrol is a candidate drug for the treatment of stroke.

**Rhein** (PubChem CID: 10168) is the primary plant metabolite in several traditional Chinese herbal medicine. It has a variety of pharmacological properties, including antioxidant, antitumor, antifibrosis, and anti-inflammation properties (Wu et al., 2020). The molecular formula of a is C_15_H_8_O_6_. Rhein effectively inhibited OS, intracellular ROS production, and protein expression associated to ferroptosis in both *in vivo* and *in vitro* MCAO models. Mechanistically, rhein counteracted OGD/R-induced damage in HT22 cells by modulating the NRF2/SLC7A11/GPX4 signaling pathway (Liu et al., 2023d). At present, there are relatively few studies on stroke from rhein, and the liver and kidney toxicity of rhein is also very high. More studies should be carried out in the future. We don't think this is a promising candidate drug.

### 5.4 Alkaloids

Alkaloids are a kind of nitrogen-containing alkaline organic compounds that exist in nature (mainly plants, but some also exist in animals). Most of them have complex ring structures. The ring primarily contains nitrogen, which exhibits significant biological activity. It is one of the important effective plant metabolites in Chinese herbal medicine. Alkaloids are an important plant metabolite of natural products with structural diversity. Currently, researchers have reported over 60 types of alkaloids.

**Berberine** (PubChem CID: 2353), a bioactive alkaloid extracted from many herbal plant species, has several pharmacological properties such as antibacterial, antidiabetic, and anticancer actions (Song et al., 2020). Berberine mitigates MCAO-induced ferroptosis, as evidenced by the upregulated expression of SLC7A11 but the reduced expression of ACSL4, TFR1, and COX2 (Wang et al., 2023c). Numerous pharmacological investigations on Berberine currently exist, and it is utilized in therapeutic practice. Consequently, we believe that Berberine is an excellent candidate for medication for the treatment of stroke.

**Dauricine** (PubChem CID: 73400) the molecular formula is C_38_H_44_N_2_O_6_. It was demonstrated in the ferroptosis model of SH-SY5Y cells that the increase of GPX4 expression by dauricine suppressed ferroptosis in SH-SY5Y cells caused by RSL3 and enhanced cell survival (Peng et al., 2022). In the C57 mouse ICH model, it was demonstrated that dauricine increased the expression of GPX4 (Peng et al., 2022). This experiment validated the therapeutic effect of dauricine on stroke both *in vivo* and *in vitro*; nonetheless, there is a deficiency of data about dauricine’s impact on stroke, necessitating further pharmacological and mechanistic investigations in the future.

### 5.5 Traditional Chinese medicine formulations

**Angong Niuhuang pill (AGNHP)** originated from *Wenbing Tiaobian* written by the febrile disease expert Wu Jutong in the Qing Dynasty. *In vivo* experiments indicated that AGNHP inhibits ROS, LPO, and Fe^2+^ accumulations in MCAO and ICH rats (Bai et al., 2024). Evidence from *in vitro* experiments shown that AGNHP mitigated ferroptosis damage caused by erastin in PC12 cells, enhanced cell survival, decreased LPO and Fe^2+^ concentrations, and boosted mRNA expressions of PPARγ, AKT, and GPX4 (Bai et al., 2024). Subsequent investigation demonstrates that AGNHP reduced the damage caused by MCAO and ICH by inhibiting ferroptosis through the activation of the PPARγ/ATK/GPX4 pathway (Bai et al., 2024).

**Di-Huang-Yin-Zi (DHYZ)** is a traditional herbal medicine employed for the prevention and treatment of neurological disorders since the Song Dynasty. The treatment with DHYZ reduced levels of ROS and MDA, thereby suppressing the expression of markers associated to ferroptosis, including Fe^2+^, SLC7A11, and GPX4 (Yang et al., 202b). The mechanism by which DHYZ reduces symptoms and improves the functional capacity of rats with poststroke depression is mostly through the suppression of ferroptosis via the P53/SLC7A11/GPX4 pathway (Yang et al., 2024b).

**Naodesheng pills (NDSP)** are commonly prescribed traditional Chinese medicines consisting of *Panax notoginseng* (Burk.) F. H. Chen, *C. tinctorius L., Crataegus pinnatifida* Bunge and *Puerariae Lobatae Radix*, and *Ligusticum chuanxiong* Hort. Both *in vivo* and *in vitro* experiments have shown that NDSP can increase the expression of GPX4, SLC7A11, and SOD while inhibiting the expression of MDA, TFR1, DMT1, ROS, and Fe^2+^ (Yang et al., 2024b). This mechanism is associated with the regulation of ferroptosis via the ERK1/2 signaling pathway (Yang et al., 2024b).

**Naotai formula (NTF)** is composed of *A. membranaceus*, *Rhizoma Chuanxiong*, *Lumbricus* and *Bombyx Batryticatus*. NTF could prevent MCAO-induced neuronal ferroptosis in rats by increasing the levels of SCL7A11, GPX4, and GSH (Lan et al., 2020). Hepcidin, BMP6, and SMADs levels were also lowered by NTF treatment, while SLC40A1 and GPX4 levels were raised (Liao et al., 2023).

**Tongluo Decoction (TLD)**, a traditional Chinese medicine prescription, has been extensively employed for the management of ischemic stroke. TLD reduced MDA, ROS and Fe^2+^ related activity and increased SOD levels (Li et al., 2024b). Compared with the MCAO group, TLD treatment significantly upregulated Nrf2, SLC7A11, FTH1, GPX4 and HO-1 levels (Li et al., 2024b). Mechanistically, TLD rescued endoplasmic reticulum stress and ferroptosis but promoted Sonic Hedgehog signaling in rats with MCAO (Hui et al., 2022).

**Xingnaojing (XNJ)** is a traditional Chinese medicinal agent used clinically for treating stroke. XNJ has been approved by the Chinese National Drug Administration. XNJ reportedly increases BBB permeability, lowers inflammation, and boosts circulation (Qu et al., 2019). Compared with the MCAO group, XNJ can reduce the expression of COX-2, TFR, and DMT1 and increase the expression of GPX4, FPN, and HO-1 (Liu et al., 2023e). Moreover, XNJ increased GPX4 levels and inhibited COX-2 and TFR protein expression after SH-SY5Y cell hypoxia (Liu et al., 2023e).

**Tongqiao Huoxue Decoction (TQHX)** mainly composed of eight botanical drugs, including *Paeoniae Radix Rubra*, *Rhizoma Chuanxiong*, *Prunus persica (L.)* Batsch, *Ziziphus jujuba Mill.*, *Zingiberis Rhizoma Recens*. TQHX is employed to improve blood circulation and eradicate blood stasis. In comparison to the MCAO group, the TQHX treatment effectively reduced the levels of MDA, Fe^2+^, ROS and ACSL4 while simultaneously increasing the expression of SOD, FTH1, and GPX4 (Ou et al., 2024).

***Salvia miltiorrhiza* (SM)** has a long history of application in China. It was first recorded in the Shennong Herbal’s classic of the Eastern Han Dynasty. SM is a commonly employed treatment for vascular diseases, particularly ischemic cardiovascular diseases. The levels of 4-HNE, ACLS4 and MDA in the penumbra of the MCAO mouse brain could be reduced by SM, which was induced by OS upregulation (Ko et al., 2023). In MCAO mice, SM treatment upregulated GPX4 and GSH and reduced FPN1 and ferritin (Ko et al., 2023).

**Paeoniae Radix Rubra (PRR)**, the root of *Paeonia lactiflora Pall*. or *Paeonia veitchii Lynch*, is extensively used in Chinese clinical practice to enhanjce blood circulation and alleviate blood stagnation. An *in vivo* study revealed that PRR enhanced the expression of GPX4, FTH1, Beclin1, LC3 II, and p-Akt in the rat of MCAO (Zhao et al., 2023b). Additionally, the *in vitro* study showed that PRR can reduce H_2_O_2_-induced HT22 cell damage by regulating cytokines like MDA, reduced GSH, and ROS, as well as by elevating the expressions of GPX4 and Beclin1 (Zhao et al., 2023b).

**Danhong injection (DHI)**, a standardized injection that contains *S. miltiorrhiza* Bunge and *C. tinctorius L*., is frequently employed to treat cerebrovascular and cardiovascular diseases. Compared with the model group, DHI increased the expression of SOD, GSH, and SATB1 and decreased the expression of MDA, TfR1, and TF (Zhan et al., 2023). Mechanism research shows that DHI inhibitors ferroptosis in ischemic stroke by regulating the SATB1/SLC7A11/HO-1 pathway (Zhan et al., 2023).

**Danlou tablet (DLT)** is composed of *Pericarpium Trichosanthis*, *Allium macrostemon* Bunge, *Puerariae Lobatae Radix*, *L. chuanxiong Hort*., *S. miltiorrhiza Bunge* [Lamiaceae; Salviae miltiorrhizae radix et rhizoma], *Paeoniae Radix Rubra*, *Alisma plantago-aquatica Linn.*, *A. membranaceus, Drynariae Rhizoma* and *Curcumae Radix*. DLT promotes blood circulation, resolves phlegm and stasis, alleviates stagnation, and dispels congestion. Both *vivo* and *vitro* studies showed that DLT markedly decreased the level of COX2 protein, GSSG, and MDA, while simultaneously increasing the levels of SLC7A11, GPX4, and GSH (Liu et al., 2024).

**Salvia miltiorrhiza Bge processed with Porcine cardiac blood (PCB-DS)** aims to enhance the brain-targeted therapy of DS through the BBB. DS shows great potential as a Chinese herbal medicine for the treatment of vascular disorders, particularly cerebrovascular disorders. PCB is a characteristic of Chinese herbal medicine, and a pharmacological reference frequently used for the treatment of brain disorders. Prior research demonstrated that PCB-DS mitigated MCAO by downregulating OS, which included reducing intracellular ROS and MDA levels (Zhou et al., 2023b). In both *vivo* and *in vitro* studies, PCB-DS modulated Fe^2+^ levels, GSH, MDA, SOD, ROS, and liperfluo to inhibit ferroptosis (Zhou et al., 202a). Further studies reveal that PCB-DS directly activated GLRX5, therefore reducing the iron-starvation response, and downregulated the SLC7A11/GPX4 signaling pathway to prevent ferroptosis (Zhou et al., 2024a).

### 5.6 Polysaccharide

Polysaccharides are essential plant metabolites of the cell wall of microorganisms, the membrane of animal cells, and higher plants. It is also closely associated with physiological functions. In recent years, there has been a growing focus on polysaccharides as a significant category of bioactive natural products. Various studies have illustrated the bioactivities of natural polysaccharides, which have resulted in their use in the treatment of various diseases.

**Neutral polysaccharide** (NPGE) is a metabolite extracted from the plant that exhibits immunomodulatory, neuroprotective, and antioxidant properties. In contrast to the MCAO group, the NPGE treatment alleviated neuronal ferroptosis by increasing GPX4 levels, decreasing ROS, MDA, and Fe^2+^ excessive accumulation, and enhancing GSH levels and SOD enzymatic activity (Zhang et al., 2024). Further mechanistic research demonstrates that NPGE reduces cerebral IR injury by inhibiting ferroptosis-mediated neuroinflammation through the NRF2/HO-1 signaling pathway (Zhang et al., 2024). The pharmacodynamic experiments of NPGE were conducted very comprehensively. In the future, small molecule and protein interaction experiments should be carried out to further confirm the target of NPGE.

**Roots of *Astragalus propinquus* Schischkin** (RAP) can raise yang qi and tonifying the spleen and lung qi, thereby facilitating urination and reducing edema. Compared with the MCAO group, RAP could increase the expression of XCT, SLC3A2, IREB2, Nrf2, HO-1, and GPX4 (Chen et al., 2022a). Both granules and injections have the same effect, but granules have the best effect. The Marker for ferroptosis in the article is too simple, with Western blot experiments being the majority. Other experiments (such as immunofluorescence) should be carried out to prove the therapeutic effect of RAP on stroke from multiple perspectives.

### 5.7 Other compounds

**Cottonseed oil (CSO)** is a vegetable oil frequently intended for the dissolution of lipid-soluble medications. Prior studies have demonstrated that CSO provides protection against intestinal inflammation, tumor metastasis, OS, and atherosclerosis (Araujo et al., 2019). Notably, current research has demonstrated its effectiveness in treating disorders of the nervous system (Sun et al., 2023). Compared to the MCAO group, CSO reduced the influx of Fe^2+^, TF, and TF receptors; upregulated anti-ferroptosis proteins (GPX4, xCT, HO1, and FTH1) while down-regulating ferroptosis-related protein ACSL4; raised GSH and SOD activity; and decreased MDA and LPO levels (Sun et al., 2023). Nevertheless, an excessively small sample size may result in erroneous conclusions. Consequently, we suggest that CSO is not an optimal candidate medication for the treatment of stroke. Nevertheless, an excessively small sample size may result in erroneous conclusions. Consequently, we suggest that CSO is not an optimal candidate medication for the treatment of stroke.

**Mastoparan M (MM)** is a peptide derived from bee venom that has biological activity and mainly affects intracellular signal transduction by acting on the cell membrane. After receiving MM therapy for 24 or 48 h, the ischemic hemisphere of MCAO mice showed a decrease in Fe^2+^ and MDA levels, but a rise in the expression of the proteins LC3B, x-CT, NRF2, and GPX4 (Sun et al., 2023). In addition, these results were confirmed in three models: OGD/R, peroxidation mediated by H_2_O_2_, and ferroptosis triggered by erastin (Sun et al., 2023). However, MM has conducted relatively few studies on stroke, and more fundamental research needs to be carried out in the future.

**Melatonin** is one of the hormones secreted by the pineal gland, which is an amine hormone produced by the pineal gland. Within the HT-22 cell model, melatonin enhanced cell proliferative capacity, decreased apoptosis, and lowered ROS generation (Sun et al., 2024b). Melatonin’s protective benefits are mediated by its inhibition of ferroptosis, an iron-dependent form of controlled cell death, via the regulation of the ACSL4/CYP1B1 pathway, according to further mechanistic investigations (Sun et al., 2024b). Melatonin lower LPO, ROS generation, and ACSL4 protein expression in MCAO mice (Sun et al., 2024b). Another study found that melatonin regulates ACSl4 ubiquitination and impacts ferroptosis by increasing MDM2 expression, which contributes to its therapeutic efficacy in stroke treatment (Ji et al., 2024). Melatonin is an over-the-counter medication in the United States and Canada, and a dietary supplement in mainland China; hence, it is not regarded as a viable pharmaceutical option for stroke treatment.

**Dl-3-n-butylphthalide** (PubChem CID: 61361, DL-NBP) is a synthetic compound extracted from celery seeds. The effectiveness and safety of the treatment have been assessed in multiple clinical trials conducted in China (Fan et al., 2021). Compared with the MCAO group, DL-NBP could significantly reduce the levels of MDA, ROS, and Fe^2+^ and increase the expression of GSH, GPx4, and SLC7A11 (Xu et al., 2023b). Further research into the mechanism revealed that DL-NBP presumably acts as a mediator in the SLC7A11/GSH/GPX4 signaling pathway to mitigate ferroptosis (Xu et al., 2023b). There have been many pharmacological studies on DL-NBP, and DL-NBP has also been applied in clinical practice. Therefore, we believe that DL-NBP is an effective candidate drug for the treatment of stroke.

**Taurine** (PubChem CID: 1123) is an amino acid converted from sulfur-containing amino acids that is widely distributed in various tissues and organs in the body. It mainly exists in the interstitial fluid and intracellular fluid in a free state. Its molecular formula is C_2_H_7_NO_3_S. The current investigation revealed a significant reduction in taurine levels in cerebrospinal fluid among patients with SAH (Liu et al., 2022b). This finding implies that administering taurine treatment after SAH may enhance neurological impairment, reduce OS, regulate iron accumulation, maintain BBB integrity, and prevent neuronal ferroptosis in the SAH model *in vivo* (Liu et al., 2022b). Thorough investigations suggest that taurine may modulate MDA levels and ROS accumulation, as well as control the expression of SLC7A11 and GPX4, and the AKT/GSK3β pathway *in vitro* (Liu et al., 2022b). Nonetheless, there exists an absence of research about the treatment of stroke by taurine. Future research should focus on more mechanism and efficacy investigations. Consequently, we do not regard taurine as a viable candidate medication for the treatment of stroke.

**Crocin** (PubChem CID: 5281233) is a hydrophilic carotenoid that is synthesized in the *Crocus sativus L.* Compared to the ICH group, the crocin-treated group significantly increased the activities of SOD and GSH-px (Wang et al., 2022b). On the other hand, crocin therapy significantly reduced the concentration of MDA (Wang et al., 2022b). The observed elevation in Fe^2+^ concentration and the upregulation of GPX4, FTH1, and SLC7A11 genes demonstrated the inhibition of ferroptosis in neuron cells by crocin (Wang et al., 2022b). Mechanistically, crocin alleviates ICH-induced neuronal ferroptosis by facilitating Nrf2 nuclear translocation (Wang et al., 2022b). While there are limited studies on the treatment of ICH by crocin, pertinent research exists regarding the treatment of ischemic stroke by crocin (Shahbaz et al., 2022). Future efficacy studies should be conducted to validate the treatment of ICH by crocin.

## 6 Clinical translation discussion

**FIGURE 5:**
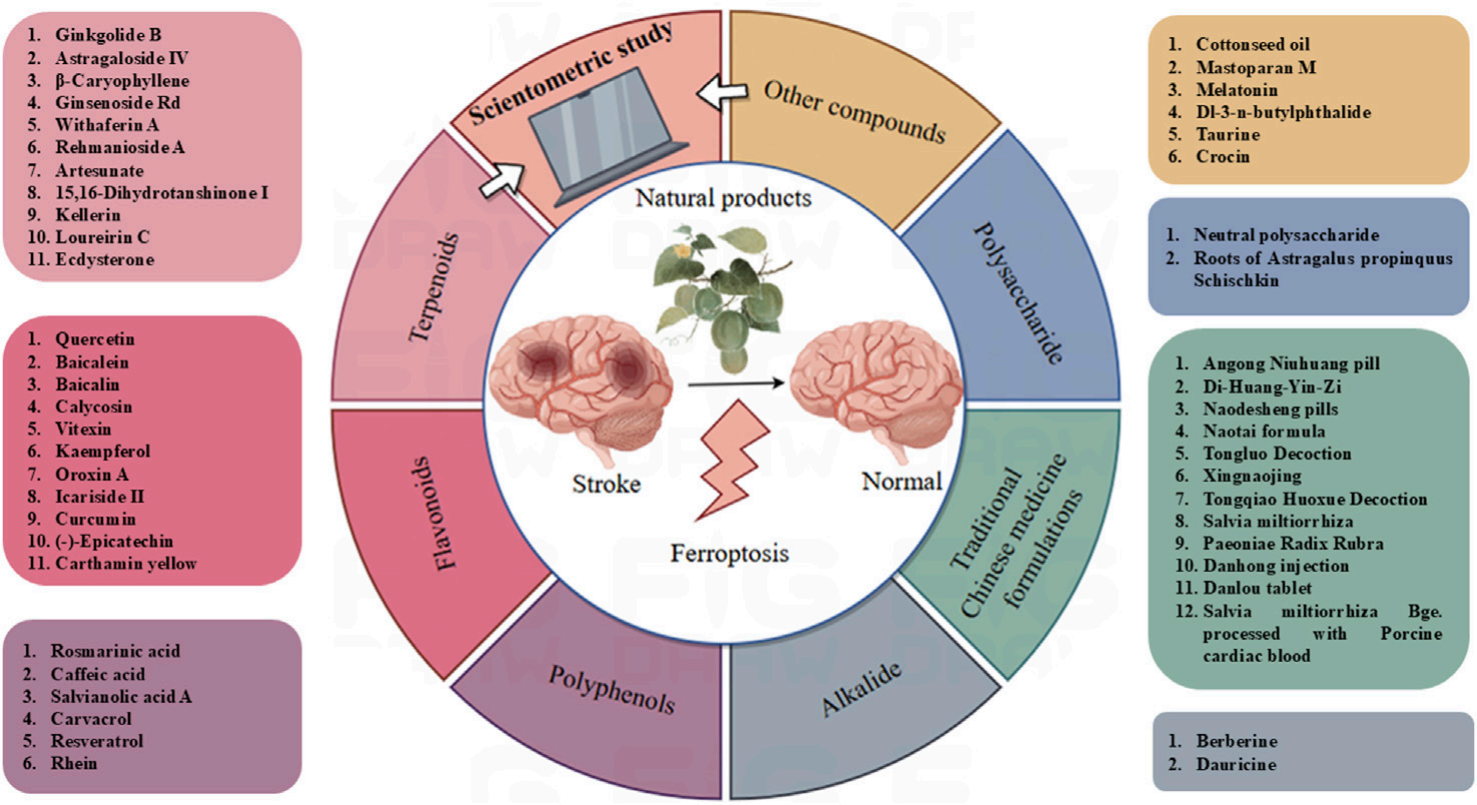
Application of natural products and herbal medicine in ferroptosis after stroke.

Compared with the previous review published by Zhou et al., we have summarized the mechanism of ferroptosis in stroke and summarized its natural products, rather than only focusing on ischemic stroke (Wang et al., 2024b). Another review systematically summarizes the status and progress of TCM in regulating various CNS diseases through the ferroptosis pathway (Zhou et al., 2024b). Its emphasis is on TCM rather than natural products. This article assesses traditional Chinese medical treatment methods, including Chinese herbal medicine, acupuncture, and moxibustion, as well as their mechanisms of action in regulating the ferroptosis pathway (Zhou et al., 2024b). In this review, we systematically summarized the mechanisms and targets of ferroptosis after stroke and summarized the related natural products (Figure 5). We aimed to provide insights into more feasible treatment schemes based on the currently proven effective natural products and Chinese medicine. Although natural products perform well as anti-ferroptosis in stroke animal experiments, there are few clinical applications. Thus, we summarized the clinical trial of the 56 natural products aimed at stroke treatment (Table 4, www.clinicaltrials.gov.). We discovered that clinical trials on stroke only used 7 natural products (Table 4).

**TABLE 4:** Clinical trials on the treatment of stroke using the above natural products.

Name	Dose	Sample size	Trial phase	Clinical trial Identifier
Ginsenoside Rd	10, 20 mg/14 days	199	Completed	NCT00591084
	20 mg/14 days	390	Completed	NCT00815763
Quercetin	—	106	Completed	NCT01376011
Rhein	—	12,000	Recruiting	NCT03157934
Salvia miltiorrhiza		46	Completed	NCT02176395
		1,900	Observational	NCT04359589
		2,200	Completed	NCT02334969
		1,503	Completed	NCT01677208
Danhong Injection	40 mL, 10 days	320	Unknown status	NCT02152280
		46	Completed	NCT02176395
		1,503	Completed	NCT01677208
Melatonin	14 mg, 90 days	140	Unknown status	NCT01863277
	10 mg, 90 days	100	Recruiting	NCT05857046
	3 mg, 14 days	80	Recruiting	NCT05247125
Dl-3-n-butylphthalide	5–10 mg, 24weeks	3,200	Recruiting	NCT05976152
	100 mL, 10 days	84	Completed	NCT02149875

From the translation of preclinical evaluation to clinical trial, model animals for stroke are relatively simple and cannot fully represent the more complex pathological features of human beings. This phenomenon may have multiple causes: 1. Regarding natural active plant metabolites, while the pharmacodynamic effects and targets are rather well-defined, research on their remains is inadequate. The study on the *in vivo* processes (absorption, distribution, metabolism, excretion, toxicokinetic) and safety assessment is insufficient, prolonging the research and development cycle of new pharmaceuticals from laboratory to clinical use; 2. The difficulty of standardizing traditional Chinese medicine. The investigations on herbal extracts and formulations reveal a complex composition, resulting in challenges in target identification, and the mechanisms involved may not be readily or thoroughly clarified. Moreover, the many technologies employed in the manufacturing of herbal extracts and formulations pose a challenge to the quality control of their active plant metabolites. 3. Disease model. Animal models struggle to accurately replicate the pathogenesis of patients, a fundamental issue that hinders the development of laboratory drugs.

Enhanced exploitation of natural products for illness treatment necessitates the development of specific methods and feasible methodologies. 1. Natural products with well-defined targets can improve mechanistic comprehension in fundamental research and promote their use in clinical applications. Biotechnological techniques, such as the cellular thermal shift assay (CETSA) or target-responsive accessibility profiling (TRAP) can be employed to identify the targets of various natural products. 2. The *in vivo* pharmacokinetic and pharmacological properties should be investigated (Tu et al., 2023). 3. Through pre-clinical research or illness prediction models, the dosage of a particular natural substance must correspond with the optimal type, stage, and timing of the disease. 4. Certain natural products encounter obstacles such as restricted water solubility, diminished bioavailability, or insufficient stability. Structural alteration, nano-delivery, or co-administration may be utilized to enhance efficacy. Utilizing multi-omics and artificial intelligence facilitates a more thorough exploration of the mechanisms behind natural products (Zhu et al., 2022b).

Preclinical models may be utilized to evaluate the efficiency of prospective therapies or procedures, acknowledging that the significance of harm and protective pathways, along with the administration of therapy, may vary between the preclinical model and people. Preclinical models can yield critical insights into the pharmacokinetics and pharmacodynamics of prospective treatments, along with potential toxicity. Critical factors must be evaluated when determining whether to forward a medication or technique from preclinical testing in stroke models to clinical trials. Effective translation necessitates diverse expertise, including basic scientists, clinical trialists, neuroimaging specialists, neurosurgeons, and rehabilitation specialists. Clinically significant outcome metrics are essential in preclinical modelling, while recognizing the intrinsic limitations of these models. Conversely, the prospective influence of a target or outcome measure within a model (together with its constraints) must be conveyed to those implementing it in clinical trials. Preliminary collaboration throughout the translational pipeline is expected to produce more resilient preclinical modelling and clinical trial design. At present, neurological impairments serve as the principal outcome measurement to assess preclinical efficacy in clinical trials. Nonetheless, possessing supplementary biomarkers or surrogate measures (e.g., imaging, inflammatory markers, brain oedema, and atrophy), particularly in relation to specific therapeutic target engagement, can be highly beneficial for comparative analysis across species and in clinical trials.

Currently, research on ferroptosis concentrates on four primary aspects: morphological feature assessment, gene expression analysis, protein level evaluation, and biochemical characteristic index measurement. Thus, the absence of definitive and authoritative standards for ferroptosis detection has emerged as a significant concern in preclinical and clinical research. Finally, both the development of natural drugs or traditional Chinese medicine and the exploration of the mechanism of stroke disease need a long way to go.

## 7 Conclusion

In this review, we summarize the natural products and herbal medicine currently employed in ferroptosis along with their mechanisms of action, highlighting the potential and challenges of clinical translation. This review is to accelerate the development of novel natural and herbal medicine treatments and to offer novel perspectives on how to treat ferroptosis in stroke.

## Glossary

ICH: Intracerebral hemorrhage
SAH: Subarachnoid hemorrhage
BBB: Blood-brain barrier
RBC: Red blood cells
HO-1: Heme oxygenase-1
CO: Carbon monoxide
TFR1: Transferrin receptor 1
STEAP3: Six-Transmembrane Epithelial Antigen of Prostate 3
LIP: Labile iron pool
SLC11A2: Solute Carrier Family 11 Member 2
LOXs: Lipoxygenases
ROS: Reactive oxygen species
NCOA4: Nuclear receptor coactivator 4
ATG5: Autophagy-related 5
ATG7: Autophagy-related 7
NFS1: Nitrogen fixation 1
HSPB1: Heat shock factor-binding Protein 1
PUFAs: Polyunsaturated fatty acids
I/R: Ischemia-reperfusion
GSH: Glutathione
MCAO: Middle Cerebral Artery Occlusion
RosA-LIP: Osmarinic acid encapsulated in liposomes
SOD: Superoxide Dismutase
MDA: Malondialdehyde
GPX4: Glutathione Peroxidase 4
ACSL4: Acyl-Co A synthetase long-chain family member 4
FTH1: Ferritin Heavy Chain 1
LDH: Lactate Dehydrogenase
Nrf2: Nuclear factor erythroid 2 related factor 2
Fto: Fat mass and obesity-associated protein
Atf3: Activation transcription factor 3
ZO-1: Zonula Occludens 1
xCT: Cystine/Glutamate Antiporter
COX2: Cyclooxygenase-2
4-HNE: 4-Hydroxynonenal
GSH-Px: Glutathione peroxidase
MMP-9: Matrix Metalloprotein 9
OS: Oxidative stress
RNS: Reactive nitrogen species
CNS: Central Nervous System
HDAC1/2: Histone deacetylases 1/2
H3K9me3: Histone H3 Lysine 9 Trimethylation
Nedd4: Neural precursor cell expressed, developmentally downregulated 4
FOXO3: Forkhead box O3
CISD2: CDGSH iron sulfur domain 2
H2S: H2S-producing enzymes
circAFF1: circular RNA AFF1
SLC7A11: Solute Carrier Family 7 Member 11
METTL3: Methyltransferase like 3
FSP1: Ferroptosis-suppressor-protein 1
Fpn: Ferroportin
IRP: Iron Regulatory Protein
Tau: microtubule-associated protein tau
LncRNA PVT1: plasmacytoma variant translocation 1
SSAT1: Spermidine/spermine N1-acetyltransferase 1
UBIAD1: UbiA prenyltransferase domain containing 1
PGE2: ProstaglandinE2
FtMt: Ferritin Mitochondrial
USP14: ubiquitin specific peptidase 14
SIRT6: Sirtuin 6
mTOR: Mechanistic Target of Rapamycin
